# Microbial metabolism influences microplastic perturbation of dissolved organic matter in agricultural soils

**DOI:** 10.1093/ismejo/wrad017

**Published:** 2024-01-10

**Authors:** Xinran Qiu, Sirui Ma, Jianrui Pan, Qian Cui, Wei Zheng, Ling Ding, Xujun Liang, Baile Xu, Xuetao Guo, Matthias C Rillig

**Affiliations:** College of Natural Resources and Environment, Northwest A&F University, Yangling, Shaanxi 712100, China; Key Laboratory of Plant Nutrition and the Agro-Environment in Northwest China, Ministry of Agriculture, Yangling, Shaanxi 712100, China; College of Natural Resources and Environment, Northwest A&F University, Yangling, Shaanxi 712100, China; Key Laboratory of Plant Nutrition and the Agro-Environment in Northwest China, Ministry of Agriculture, Yangling, Shaanxi 712100, China; College of Natural Resources and Environment, Northwest A&F University, Yangling, Shaanxi 712100, China; Key Laboratory of Plant Nutrition and the Agro-Environment in Northwest China, Ministry of Agriculture, Yangling, Shaanxi 712100, China; College of Natural Resources and Environment, Northwest A&F University, Yangling, Shaanxi 712100, China; Key Laboratory of Plant Nutrition and the Agro-Environment in Northwest China, Ministry of Agriculture, Yangling, Shaanxi 712100, China; College of Natural Resources and Environment, Northwest A&F University, Yangling, Shaanxi 712100, China; Key Laboratory of Plant Nutrition and the Agro-Environment in Northwest China, Ministry of Agriculture, Yangling, Shaanxi 712100, China; College of Natural Resources and Environment, Northwest A&F University, Yangling, Shaanxi 712100, China; Key Laboratory of Plant Nutrition and the Agro-Environment in Northwest China, Ministry of Agriculture, Yangling, Shaanxi 712100, China; College of Natural Resources and Environment, Northwest A&F University, Yangling, Shaanxi 712100, China; Key Laboratory of Plant Nutrition and the Agro-Environment in Northwest China, Ministry of Agriculture, Yangling, Shaanxi 712100, China; Institut für Biologie, Freie Universität Berlin, Berlin 14195, Germany; Berlin-Brandenburg Institute of Advanced Biodiversity Research, Berlin 14195, Germany; College of Natural Resources and Environment, Northwest A&F University, Yangling, Shaanxi 712100, China; Key Laboratory of Plant Nutrition and the Agro-Environment in Northwest China, Ministry of Agriculture, Yangling, Shaanxi 712100, China; Institut für Biologie, Freie Universität Berlin, Berlin 14195, Germany; Berlin-Brandenburg Institute of Advanced Biodiversity Research, Berlin 14195, Germany

**Keywords:** FT–ICR–MS, microplastics, dissolved organic matter, microbiome, metabolic pathway

## Abstract

An estimated 258 million tons of plastic enter the soil annually. Joining persistent types of microplastic (MP), there will be an increasing demand for biodegradable plastics. There are still many unknowns about plastic pollution by either type, and one large gap is the fate and composition of dissolved organic matter (DOM) released from MPs as well as how they interact with soil microbiomes in agricultural systems. In this study, polyethylene MPs, photoaged to different degrees, and virgin polylactic acid MPs were added to agricultural soil at different levels and incubated for 100 days to address this knowledge gap. We find that, upon MP addition, labile components of low aromaticity were degraded and transformed, resulting in increased aromaticity and oxidation degree, reduced molecular diversity, and changed nitrogen and sulfur contents of soil DOM. Terephthalate, acetate, oxalate, and L-lactate in DOM released by polylactic acid MPs and 4-nitrophenol, propanoate, and nitrate in DOM released by polyethylene MPs were the major molecules available to the soil microbiomes. The bacteria involved in the metabolism of DOM released by MPs are mainly concentrated in *Proteobacteria*, *Actinobacteriota*, and *Bacteroidota*, and fungi are mainly in *Ascomycota* and *Basidiomycota*. Our study provides an in-depth understanding of the microbial transformation of DOM released by MPs and its effects of DOM evolution in agricultural soils.

## Introduction

The increasing amount of plastic wastes in the soil environment is a global problem, posing great threats to soil ecosystems and food security [1-3]. The improper use and recycling of plastic products lead to further fragmentation of plastics into microplastics (MPs; <5 mm) in the global terrestrial environment, which makes soil a main reservoir of MPs, especially the agroecosystems [4-6]. MPs inevitably cause microbiome responses in soil [7]. In terms of their effects on soil microbial activity, polypropylene microplastics had positive effects, while polyacrylic, polyester (PE), and polystyrene (PS)-MPs showed negative effects [8-10]. However, a recent study found that biodegradable particles promoted soil microbial functional diversity, along with no effect of PE microplastics (PE-MPs) and PS-MPs on the functional diversity of soil microbiomes [11]. The different effects of MPs on soil microbial activity observed are likely attributed to various types of polymers, degree of aging, and concentrations used in previous studies. Furthermore, most studies neglected the effect of MPs on soil chemical compositions, for instance, soil dissolved organic matter (DOM), at the molecular level and their relation with the changes of soil microbiome [9, 12, 13]. Therefore, it is difficult to draw general conclusions about the bioturbation of MPs in soils.

Commercial plastics often contain various types of additives that often contain certain biotoxic compounds, and the structural damage and aging of MPs under the influence of environmental factors such as ultraviolet (UV) light and weathering can accelerate the release of these substances to form an important part of DOM released by MPs-DOM [14-16]. Dissolved organic matter released by microplastics (MPs-DOM) can exert toxic effects on marine animals and sludge-digesting microbiota [17-19]. Studies also shown that MPs-DOM could change the composition of natural organic matter in the marine and freshwater ecosystem and fuel the growth of bacteria [20, 21]. Hitherto, most studies focused on the examination of MPs-DOM and their effects on the aquatic organisms, and the transformation processes and underlying mechanisms of MPs-DOM in the soil environment remain poorly understood [22]. The processes and how soil DOM, an important carbon source in agroecosystems, are altered by MPs at the molecular level have rarely been explored, and the contribution of soil microbiomes in this process remains to be understood [11, 12].

In this study, PE, the main component of agricultural land film, and polylactic acid (PLA), a widely used biodegradable plastic, were chosen as representative MPs in agricultural soil systems [23, 24]. PE-MPs and PLA-MPs with different physicochemical properties were added to the soil and incubated for 100 days to (i) reveal the composition and structural changes of soil DOM after MPs addition, (ii) explore the changes in soil microbial community composition after MPs application, and (iii) determine the transformation of MPs-DOM by soil microbiomes and its relation to the changes of soil DOM. This study helps to elucidate the perturbation effects of MPs on agricultural soil ecology and DOM evolution.

## Materials and methods

### Chemicals and materials

The surface agricultural soils (0–20 cm) collected from Yangling District, Shaanxi Province, China (34°20′N, 108°04′E) were air dried, homogenized, and sieved with a 2 mm sieve before use (The detailed properties of the original soil and the background MPs are in Supplementary Text S1 and Supplementary Fig. S1) [25]. PE-MP and PLA-MP powders were purchased from Sinopec Beijing, and both were sieved with 200 mesh (particle size of ~75 μm). Some PE-MPs were irradiated with UV light (300 W, 270 μw/cm^2^) in air for 5 and 10 days, respectively. The SEM images of the virgin and aged MPs used in this study are shown in Supplementary Fig. S2.

### Soil incubation experiments

To examine the effects of DOM derived from different types and concentrations of MPs, nine treatments were designed as follows: (a) unamended soil (CK), (b) soil mixed with 0.5% (w/w) virgin PE-MPs (0.5PE), (c) soil mixed with 1.5% virgin PE-MPs (1.5PE), (d) soil mixed with 0.5% PE-MPs aged for 5 days, (e) soil mixed with 1.5% PE-MPs aged for 5 days (1.5PE5d), (f) soil mixed with 0.5% PE-MPs aged for 10 days, (g) soil mixed with 1.5% PE-MPs aged for 10 days (1.5PE10d), (h) soil mixed with 0.5% virgin PLA-MPs, and (i) soil mixed with 1.5% virgin PLA-MPs (1.5PLA). All treatments were done in triplicates and mixed thoroughly before incubation using sterilized shovel. Opaque pots were used as experimental units and filled with 10 kg of sieved soil and corresponding amounts of MPs (Supplementary Fig. S3). The concentrations (0, 0.5, and 1.5%) of MPs were equivalent to 0, 10, and 30 t/ha. The 0.5% MPs concentration is close to that observed in many agricultural soils, while the high concentration level of 1.5% MPs may be commonly reached at future contamination levels [26-28]. Soil moisture was maintained at about 60% of the soil field capacity by watering every 5 days throughout the trial [29, 30]. Soil samples were collected at 25, 50, 75, and 100 days and stored at −80°C for further use.

### Extraction and spectral determination of soil dissolved organic matter

The content of dissolved organic carbon (DOC) in DOM extracted from soil (see Supplementary Text S2 for detailed extraction method) was determined using a total organic carbon analyzer (Shimadzu, Japan), with a detection limit of 4 μg/l [31]. Ultraviolet absorbance at 254 nm (UV_254_) was measured with an ultraviolet spectrophotometer (GENESYS 10S UV-vis, Thermo Fisher, USA) [32]. The UV_254_ was divided by DOC to compute the values of specific ultraviolet absorbances at 254 nm (SUVA_254_, see Supplementary Text S3 for details). 3D-excitation emission matrix (3D-EEM) of extracted soil DOM was obtained by fluorescence spectrophotometer (RF-6000, Shimadzu, Japan), and the data were modeled by parallel factor analysis (PARAFAC) to obtain organic fluorescence fraction results (details provided in Supplementary Text S4 and S5) [33-36].

### Mass spectrometry characterization of soil dissolved organic matter

For the qualitative detection of the molecular composition of MPs-DOM, quadrupole time-of-flight (Q-TOF) mass spectrometry (Agilent 1290-6545) was used for the detection of MPs-DOM, as detailed in Supplementary Text S6 [37, 38]. Combined with the results of the metabolites of MPs incorporated into the soil, the MPs-DOM molecular components that can be used by microbiomes were screened by kyoto encyclopedia of genes and genomes (KEGG) metabolic pathway analysis (https://www.genome.jp/kegg/). The molecular composition of soil DOM was quantitatively determined by Electrospray ionization Fourier transform ion cyclotron resonance mass spectrometry (ESI–FT–ICR–MS). Before ESI–FT–ICR–MS analysis, samples were desalted and concentrated by solid phase extraction (Agilent Bond Elut PPL, 500 mg, 6 ml), as described in Supplementary Text S7 [32]. Samples were detected by 15T Bruker Apex Ultra FT–ICR mass spectrometer (Bruker Daltonics, Germany). The detailed detection and calculation methods are given in Supplementary Texts S8 and S9, respectively.

### Soil DNA extraction and sequencing

All the DNA were obtained from samples using the Power DNA Isolation Kit (MO BIO Laboratories). DNA quantity and quality were evaluated by the ratios of 260 nm/230 nm and 260 nm/280 nm. DNA was then stored at −80°C. The collected soil samples were subjected to high-throughput sequencing analysis of bacteria and fungi (Illumina Novaseq platform of the Biomarker Technologies Corporation, Beijing, China). For bacteria, primers (Forward primer: ACTCCTACGGGAGGCAGCA; Reverse primer: GGACTACHVGGGTWTCTAAT) were used to amplify the V3–V4 region of the rRNA gene; for fungal ITS rDNA, the highly variable ITS1 region was amplified using primers containing the sequence CTTGGTCATTTAGAGGAAGTAA and the reverse primer containing the sequence GCTGCGTTCTTCATCGATGC [25]. The total volume of polymerase chain reaction (PCR) was 50 μl, including 0.2 μl of Q5 High-Fidelity DNA Polymerase, 10 μl of Buffer, 10 μM of each primer, 10 μl of High GC Enhancer, 1 μl of dNTP, and 60 ng of genome DNA. Thermal cycling conditions were as follows: an initial denaturation for 5 min at 95°C, followed by 15 cycles for 1 min at 95°C, 50°C for 1 min, and 72°C for 1 min, with a final extension for 7 min at 72°C. The PCR products from the first amplification were purified by VAHTS DNA Clean Beads. The purified product was subjected to secondary amplification in a 40 μl reaction system consisting of 8 μl dd H_2_O, 20 μl 2 × Phusion HF MM, 10 μM of each primer, and 10 μl PCR products from the first step. Thermal cycling conditions were as follows: an initial denaturation for 30 s at 98°C, followed by 10 cycles for 10 s at 98°C, 65°C for 30 s, and 72°C for 30 s, with a final extension for 5 min at 72°C. Total PCR products were quantified by Quant-iT dsDNA HS Reagent.

### Untargeted metabolomics analysis

A certain amount of collected soil samples were extracted using extraction solutions composed of methanol, acetonitrile, water, and 2 mg/L internal standard for the examination of microbial transformation of soil DOM during incubation. The metabolomics analysis was performed using a Waters Acquity I-Class PLUS UPLC tandem with a Waters Xevo G2-XS QTOF high-resolution mass spectrometer. Detailed extraction and detection protocols are presented in Supplementary Text S10.

### Bioinformatics analysis

After soil DNA extraction and sequencing, the raw sequences were assembled for each sample according to a unique barcode after removing the adaptor and primer sequences, and the raw tags were filtered for quality [39, 40]. Briefly, raw sequences with more than three consecutive low-quality base calls (Phred quality score ≤ 19) were truncated at the position where their quality began to drop, and only reads with >75% consecutive high-quality base calls and no ambiguous characters were retained for further analyses. The sequences were compared with the reference database (Gold database) using the UCHIME algorithm by Usearch to detect chimeric sequences and the chimeric sequences were then removed. Sequence analysis was performed using UPARSE (v. 7.0.1001) [41]. After discarding the singletons, sequences with ≥97% similarity were assigned to the same operational taxonomic units (OTUs). For each representative sequence, the SILVA (v. 138.1) and UNITE (v. 9.0) databases were used to annotate the taxonomic information. Samples were rarefied at 53 594 and 2895 sequences per sample for subsequent bacterial and fungal community analyses, respectively, to correct for sampling effort (number of analyzed sequences per sample).

### Statistical analysis

The data obtained by sampling soils with the same MPs incubation conditions at different times were used as a group for network construction. Network construction was based on the “ggClusterNet” (v. 0.1.0) package in R. Correlations among soil microbiome under the influence of MPs and with soil DOM were estimated using spearman correlation analysis (*r* > 0.8, *P* < .05). The network was visualized using the interactive Gephi platform (https://gephi.org). The network properties are based on the “net_properties.2” function of “ggClusterNet” and the network module is calculated based on the “cluster fast greedy” algorithm. Metabolic pathways were predicted by PICRUSt2 in R. The possible contribution of bacteria and fungi in the production of relevant metabolites was assessed using a Random Forest model, which was constructed primarily on an R language platform using the “randomForest” (v. 4.7.1.1) package. Ternary phase diagram, bubble diagram, and Venn diagram were drawn by “ggtern “(v. 3.4.0), “ggplot2”(v. 3.4.0), and “venndiagram”(v. 1.7.3), respectively.

## Results

### Addition of microplastics changes the characteristics of soil dissolved organic matter

#### Microplastics increase dissolved organic carbon contents and aromaticity of soil dissolved organic matter

**Figure 1:**
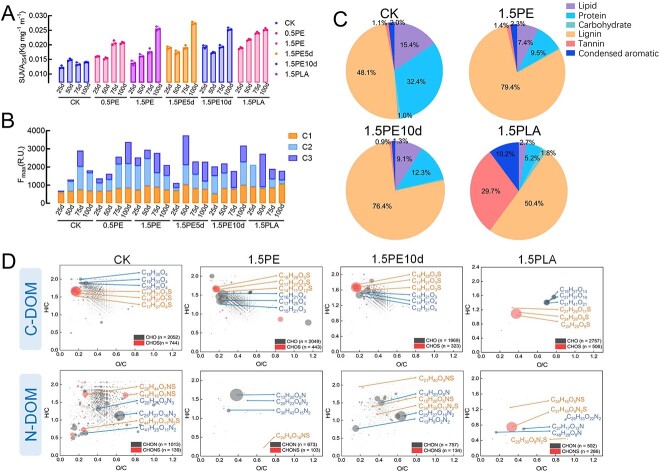
Changes in the spectral properties and molecular compositions of soil DOM after incorporation of MPs with different characteristics into the soil; (A) changes in the SUVA_254_ index (error bars represent standard deviation, *n* = 3); (B) changes in the three components, C1 (humic-like), C2 (protein-like), and C3 (fulvic-like), of soil DOM in EEM-PARAFAC; (C) average relative abundance of different organic carbon fractions in van Krevelen plots of soil DOM in control (CK), 1.5PE, 1.5PE10d, and 1.5PLA soil DOM; (D) chemical formulae of soil DOM after incubation for 100 days; all chemical formulae were divided into N-containing (N-DOM) and N-free (C-DOM) molecules; the size of the circle in Panel D represents the relative intensity of each compound along; CK, 0.5PE, 1.5PE, 1.5PE5d, 1.5PE10d, and 1.5PLA indicate treatments with no plastics, 0.5% PE, 1.5% PE, 1.5% PE aged for 5 days, 1.5% PE aged for 10 days and 1.5% PLA.

The carbon content fluctuation of soil DOM after incorporation of MPs into soil was preliminarily analyzed by DOC and UV_254_, while SUVA_254_ obtained via dividing UV_254_ by DOC is widely used to evaluate the aromatic content of DOM (Fig. 1A and Supplementary Fig. S4) [42]. The results showed that with the addition of plastics, both biodegradable and nonbiodegradable MPs resulted in an overall increase in the carbon content and the accumulation of aromatic substances in the soil DOM. The 3D-EEM spectral analysis results of soil DOM were further analyzed by PARAFAC, and three fluorescence components were identified, including humic-like (C1), protein/phenol-like (C2), and fulvic-like (C3) (Supplementary Figs S5 and S6) [43-45]. After the addition of MPs, C1 fraction of soil DOM showed little change, whereas C2 and C3 were significantly decreased and increased, respectively (Fig. 1B). This indicated that the protein/phenol-like components were consumed upon the effect of MPs, and most of the increased organic matter was fulvic-like compounds. The aged PE group showed greater intensity of C1 and C2 compared to the unaged PE-MPs group, and the PLA-treated group also had a greater effect on soil fluorescence composition [35, 46]. The correlation indices obtained by 3D-EEM showed that the incorporation of MPs led to an increase in the soil humification, especially the incorporation of PLA-MPs (Supplementary Fig. S7) [47-49].

#### Microplastics decrease molecular diversity of soil dissolved organic matter

The molecular composition of DOM extracted from soil after incorporation of CK, 1.5PE, 1.5PE10d, 1.5PLA for 100 days was identified by FT–ICR–MS, and the data were depicted in van Krevelen diagrams (Supplementary Fig. S8 and Supplementary Table S1) [29, 50, 51]. When MPs were added to the soil, a sharp decrease in the number of molecules allocated to protein-like, carbohydrate-like, and lipid-like regions was detected, especially in the soil DOM of PLA-MPs, followed by virgin PE-MPs and aged PE-MPs (Fig. 1C) [30, 52]. The contents of lignin-like molecules and condensed aromatic molecules were relatively abundant and increased in the DOM of PLA-MPs soil samples, and the content of related substances in soil samples with PE-MPs added was slightly increased. The lignin-like substances and condensed aromatic substances in the aged PE-MPs soil samples were slightly decreased. After adding MPs, both the double bond equivalent index (DBE_w_) and the modified aromatic index (AI_w_) showed that the unsaturation and aromaticity of soil DOM increased most significantly with the addition of PLA-MPs, while soil samples with aged PE-MPs were higher than those with PE-MPs (Supplementary Table S2) [53, 54]. FT–ICR–MS spectrum analysis in broadband mode (m/z 100–1000) showed that the addition of MPs led to a general increase in the molecular weight of soil DOM, and the PLA-MPs treatment was more pronounced (Supplementary Fig. S9) [50].

The molecules detected from the soil DOM were categorized into four types: carbon, hydrogen, and oxygen atoms only (CHO); carbon, hydrogen, oxygen, and nitrogen atoms only (CHON); carbon, hydrogen, oxygen, and sulfur atoms only (CHOS); carbon, hydrogen, oxygen, nitrogen, and sulfur atoms only (CHONS). The relative abundance of CHO, CHON, CHOS, and CHONS compounds in the four soil DOM compositions indicated that the soil DOM in the treatment group contained more CHO compounds than those in the control group, especially in the1.5PLA treatment followed by the 1.5PE10d and 1.5PE5d treatment (Fig. 1D). This was supported by the relative higher intensities of DOM molecules containing more oxygen atoms in the 1.5PLA treatment, indicating that PLA-MPs were more likely to promote the oxidation of organic matter in the soil than PE-MPs (Supplementary Fig. S10 and Supplementary Fig. S11) [10, 30, 55]. In addition, the relative abundance of CHONS compounds in the MPs-treated soil was higher than that in the control soil, with the most obvious change in PLA-MPs-treated soil samples followed by aged PE-MPs, indicating that biodegradable MPs and aged MPs can enhance the accumulation of heteroatoms (Fig. 1D) [56]. By dividing soil DOM into two different components, macromolecular carbonaceous soil DOM (C-DOM) and nitrogenous soil DOM (N-DOM), it was found that the diversity of molecular formula composition of C-DOM and N-DOM decreased after the addition of MPs, with the diversity of soil DOM composition decreasing most obviously in the 1.5PLA treatment (Fig. 1D) [32]. After the incorporation of PLA-MPs, the distribution of sulfur was relatively uniform in C-DOM and N-DOM, and the molecular formula species with high carbon and high oxygen content dominated, especially in N-DOM. In the 1.5PE10d treatment, the molecular formula with sulfur in N-DOM was greatly reduced, but this improved with the aging of PE-MPs. In the control group, the molecular formula of N-DOM containing 2–3 N atoms dominated, but the nitrogen atoms in the molecular formula of DOM decreased after the addition of MPs.

### Response of microbial community to microplastic incorporation into soil

**Figure 2:**
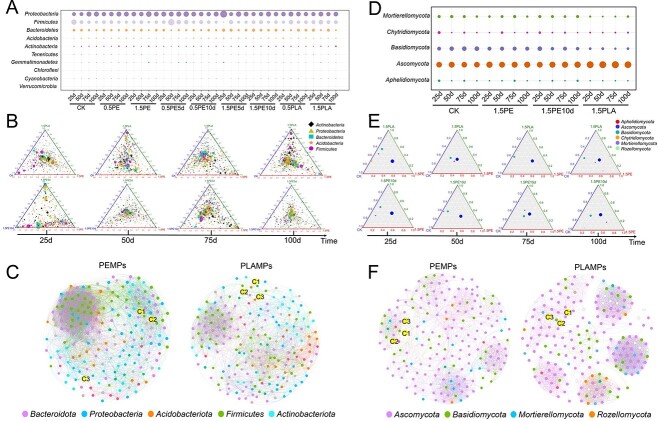
Soil microbiome diversity and co-occurrence network under the influence of MPs; mean relative abundance of bacterial (A) and fungal (D) taxa at the phylum level under the influence of different MPs; the relative abundance is indicated by the size of each circle; ternary plots of bacterial (B) and fungal (E) communities under the influence of different MPs, with edges and vertices indicate different treatments respectively and points with different shapes indicate different microbiomes at the phylum level; the data are shown in Supplementary Tables S3 and S4; co-occurrence network of bacterial (C) and fungal (F) communities under the influence of different MPs; networks are colored according to the classification of microbiomes at the phylum level, connections indicate strong and significant correlations (*P* < .05), and elliptical partitions of different colors indicate the modular distribution in the co-occurrence network; the bigger circles in the network diagram are humic-like (C1), protein/ phenal-like (C2), and fulvic-like (C3).

Our study found that MPs negatively affected the abundance and diversity of soil microbiomes, which deepened on the aging of plastics, and the incorporation of biodegradable plastics had a more pronounced effect on soil microbial abundance and diversity than nondegradable plastics (Fig. 2A and B and Supplementary Fig. S12). At the bacterial phylum level, the ternary and matrix bubble diagram show that, compared with the CK, *Actinobacteria* and *Firmicutes* gradually shifted and accumulated in 1.5PE and 1.5PLA, in which the shift in 1.5PLA was more obvious. Furthermore, *Proteobacteria* predominated in the unaged plastic treatment group, whereas *Bacteroidetes* dominated in the aged plastic treatment group (Fig. 2A and B) [25, 29]. At the fungal phylum level, the relative abundance of *Ascomycota* increased with the addition of PLA-MPs and was more affected by plastic aging. The addition of all MPs decreased the relative abundance of *Basidiomycota*, *Chytridiomycota*, *Mortierellomycota*, and *Rozellomycota*, with the inhibition by PLA-MPs, compared to that of nonbiodegradable plastics (Fig. 2D and E). At the genus level, *Auricularia* (*Basidiomycota* phylum), *Fusarium* (*Ascomycota* phylum), and *Mortierella* (*Mortierellomycota* phylum) were more affected by the admixture of MPs (Supplementary Fig. S13).

Analysis of potential microbial interaction patterns between the different MP treatment groups using co-occurrence network approach revealed a high degree of modularity in the topological networks of all soil microbiota, suggesting that the soil microbiome remained structurally and functionally stable after MPs incorporation (Fig. 2C and F) [57, 58]. Higher node and edge numbers, and average degree (AD) are often used to assess the complexity of a soil microbial network, with higher node and edge numbers and smaller centralization betweenness representing greater network complexity [59, 60]. The MP-treated soil microbiomes had more complex bacterial network patterns, especially the PLA-MP-treated group higher than the subnetworks of the CK and PE-MP-treated groups, implying that the PLA-treated soil bacterial networks were more functional and at the same time more fragile (Supplementary Tables S5 and S6). However, the addition of MPs made the network complexity slightly lower for the fungal community. To investigate the relationship between microbiome and soil fluorescence fractions, PARAFAC results of microbiome and soil fluorescence fractions were correlated (Fig. 2C and F). It was found that among the bacterial composition of PE-MPs incorporated into soil, C1 was mainly associated with *Firimicutes* and *Proteobacteria*, while C3 was mainly associated with *Acidobacteria* and *Bacteroidota*. In the PLA-MP-bacteria group, C1 was associated with *Proteobacteria* only, while C3 was mainly associated with *Actinobacteriota* and *Acidobacteria*. However, C2 was independently present in the bacterial network. In the fungal group, *Ascomycota* showed higher correlations with C1, C2, and C3 compared to other fungal groups.

### Metabolomic evidence supports microbial degradation of dissolved organic matter released by microplastics in soil

**Figure 3:**
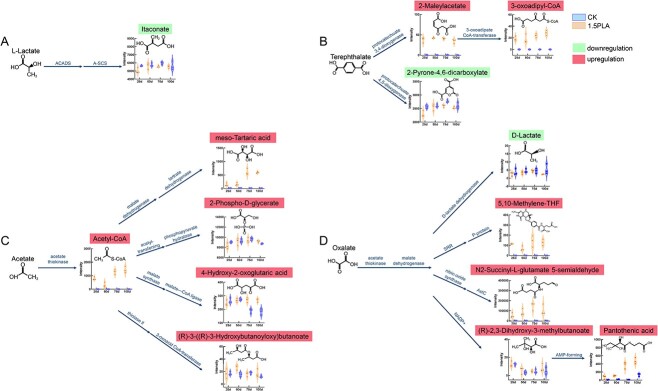
Microbial metabolic pathways of PLAMPs-DOM upon entry into the soil; the bioavailable fraction in PLAMPs-DOM was mainly L-lactate (A), terephthalate (B), acetate (C), and oxalate (D); violin plots represent the abundance of microbial metabolic MPs-DOM products; the abundance of dominant genes in metabolic pathways is shown in Supplementary Fig. S15; error bars represent one standard deviation, *n* = 3.

Our main objective is to monitor the chemical transformation of MPs–DOM by soil microbiota to find evidence of the involvement of the soil microbiome in altering soil DOM when soil is contaminated by MPs. Molecular components of PE–MPs–DOM and PLA–MPs–DOM with possible bioavailability were localized by Q-TOF combined with KEGG analysis (Supplementary Table S7). Using metabolomic product analysis and PICRUSt2 metabolic function prediction, we identified possible bacterial metabolic pathways for MPs-DOM in soil and the changes in the abundance of relevant metabolites over time (Fig. 3, Supplementary Figs S14–S16). Fungi were screened at the phylum and genus level for association with target metabolites using correlation analysis.

**Figure 4:**
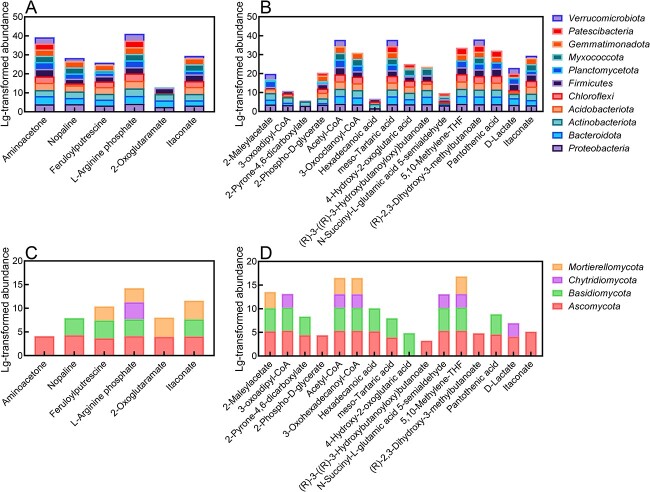
Normalized abundance of major microbiome members involved in MPs-DOM metabolism at the phylum level, including bacterial abundance (A) and fungal abundance (C) in the PEMPs-treated group and bacterial abundance (B) and fungal abundance (D) in the PLAMPs-treated group; normalized abundances were obtained by LOG transformation of bacterial and fungal abundances at the phylum level; by ranking the abundance of the microbiome with the related metabolic function genes, the microbiome (at the genus level) with the top 10 abundances was the dominant microbiome.

The main substances involved in microbial metabolism in PE–MPs–DOM are 4-nitrophenol, propanoate, and nitrate (Supplementary Fig. S14). Microbiomes metabolized propanoate mainly to itaconate through the intermediate product propanoyl-CoA and metabolized 4-nitrophenol to 2-oxoglutaramate (Supplementary Fig. S14). These metabolites were downregulated in the treatment group, which may be due to the reduction of related regulatory genes in the treatment group compared to the CK group (Supplementary Fig. S16). The bacteria that played a major role in this process at the phylum level were *Proteobacteria, Acidobacteriota*, and *Bacteroidota* (Fig. 4A and Supplementary Fig. S18). Among the fungi that may be involved in this process, *Penicillium* (*Ascomycota* phylum), *Trichoderma* (*Ascomycota* phylum), and *Linnemannia* (*Mortierellomycota* phylum) are predominant at the genus level (Fig. 4C and Supplementary Fig. S17). Nitrate was metabolized mainly to aminoacetone, which was downregulated, and nopaline, feruloyl putrescine, anserine, L-arginine phosphate were upregulated. Soil bacteria treated with PLA-MPs carried more relevant regulatory genes (Fig. 4A and C and Supplementary Fig. S17).

The main substances of PLA–MPs–DOM that can be metabolized by microbiomes are terephthalate, acetate, oxalate, and L-lactate (Fig. 3). Among them, the composition with the most abundant metabolic pathway and generally the highest product abundance was acetate, and the metabolites of which generally contained more oxygen atoms, suggesting that the metabolism of acetate may have contributed to the increase in oxygenated organic matter in the soil. By PICRUSt2 predictive analysis, we found that this metabolic pathway may be mainly present in *Proteobacteria*, *Actinobacteriota*, *Bacteroidota*, and *Chloroflexi* in bacteria, and in *Thelonectria* (*Ascomycota* phylum), *Fusarium* (*Ascomycota* phylum), and *Auricularia* (*Basidiomycota* phylum) in fungi (Fig. 4B and D and Supplementary Fig. S17). In particular, the content of 2-phospho-d-glycerate, one of the acetate metabolites, contained an overall 1.2–800-fold higher content than most metabolites, with a higher abundance of phosphorus atoms. Oxalates without nitrogen atoms may be metabolized by bacteria to form *N*-succinyl-l-glutamate 5-semialdehyde and 5,10-methylene and pantothenic acid containing nitrogen atoms. Predictions suggest that the dominant bacterial groups for this process may be *Bacteroidetes, Acidobacteriota*, and *Proteobacteria*. Whereas the metabolism of terephthalate and L-lactate may be related to *Proteobacteria, Actinobacteriota*, and *Planctomycetota* at the bacterial phylum level (Fig. 4B and Supplementary Fig. S18). In fungi, the metabolic pathway for PLA–MPs–DOM may be associated with *Thelonectria* (*Ascomycota* phylum), *Fusarium* (*Ascomycota* phylum), and *Auricularia* (*Basidiomycota* phylum) at the genus level (Fig. 4D and Supplementary Fig. S17).

To further discuss the contribution of bacteria and fungi in the metabolism of MPs-DOM soil, a random forest analysis was performed at the genus level for the microbiome involved in the metabolism of MPs-DOM (Supplementary Fig. S19). The results suggest that fungi and bacteria have different contributions to the production of various metabolites. Overall, bacteria may contribute more to MPs-DOM metabolism compared to fungi (Supplementary Fig. S20).

### Co-occurrence network analysis of microbiomes involved in the soil metabolism of dissolved organic matter released by microplastics

**Figure 5:**
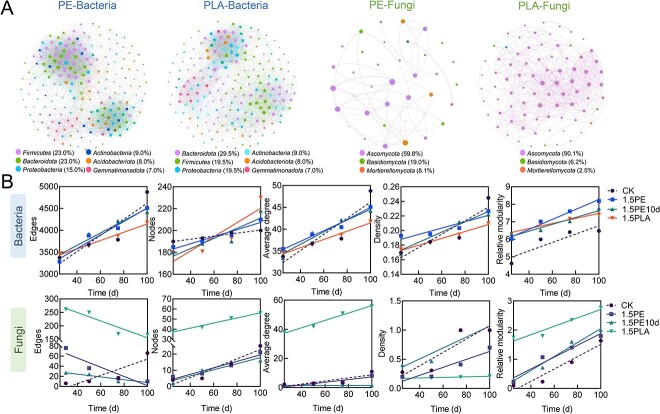
(A) Co-occurrence network of microbiome involved in the transformation process of MPs-DOM in soil; the network nodes represent the abundance of microbiomes involved in MPs-DOM metabolism; (B) changes in important parameters of the microbial co-occurrence network at 25, 50, 75, and 100 days of sampling.

To infer the interrelationships among the microbiomes involved in MPs-DOM metabolism, we constructed four networks (PE-Bacteria, PLA-Bacteria, PE-Fungi, PLA-Fungi), all of which had a high degree of positive correlation, suggesting that the taxa tend to coexist (Fig. 5). For microbiomes involved in PLA–MPs–DOM soil metabolism, the average path length (APL) and network diameter (ND) of the observed networks were significantly lower than those involved in PE–MPs–DOM soil metabolism. APL (the path is the distance between any two nodes in a network) reflects the degree of separation between individual nodes in a network, and shorter APL may be associated with better microbial cooperation, more communication, and intermediate transportation [61]. The maximum distance between any two nodes in a network is called ND. In general, soil networks with shorter ND and APL indicate higher complexity [62]. This suggests that the distance between two nodes in the microbial network of PLA–MPs–DOM metabolism connects to all other nodes through very short paths, favoring the rapid transfer of information compared to the PE–MPs–DOM treatment group (Supplementary Table S8). However, for microbial communities metabolized by PE–MPs–DOM soils, both centralization closeness, which reflect the speed of information transfer between microbiomes, and relative modularity (RM), which reflect the division of resources and the formation of subcommunities, were higher than in the PLA–MPs–DOM group, indicating that there were more highly interconnected (clustered) nodes in the soil microbial network involved in PE–MPs–DOM metabolism, which not only had stronger clustering, but also more pronounced modular aggregation of microbiomes (Supplementary Table S8) [63, 64]. In the bacterial metabolism, the co-occurrence network of PE–MPs–DOM treatment group had a higher AD, while the AD of the related fungal co-occurrence network was higher for PLA-MPs treatment. The relevant properties of the co-occurrence network also varied over time (Fig. 5B). Both the edges and nodes of the bacterial network increased with time, indicating that the bacteria involved in MPs-DOM metabolism in soil will become more and more deeply associated. The number of edges in the fungal network is decreasing, but the number of nodes representing the number of fungal OTUs tends to increase over time. Overall, the AD, RM, and density of the co-occurrence network of microbiomes involved in MPs-DOM soil metabolism increased with time, suggesting that the nodes in the co-occurrence network were more aggregated and complex.

The abundance of *Proteobacteria*, *Acidobacteriota*, *Actinobacteriota*, and *Bacteroidota* among the bacteria involved in the metabolism of MPs-DOM is higher; however, *Firmicutes* show more nodes in the network diagram and had greater mean values, so it can be inferred that *Firmicutes* may be an intermediate bacterium in the soil metabolism of MPs-DOM (Fig. 4 and Supplementary Fig. S21). Among the fungi, both *Ascomycota* and *Basidiomycota* play important roles in MPs-DOM metabolism.

## Discussion

### Utilization pathways of the soil microbiome for dissolved organic matter released by microplastics

It is now well established that MPs and MPs-DOM have an important impact on the soil properties themselves due to plastic pollution caused by the development of industrial agriculture, and that the soil microbiome plays an important role in the transformation and utilization of MPs in the context of soil pollution [22, 43, 65]. Therefore, it is important to understand how soil microbial communities are affected by agricultural MPs and to explore how the key MPs-DOM affects DOM properties of agricultural soils and the main utilization pathways of microbiomes. Until now, most previous studies have focused on the α- and β-diversity patterns of soil microbiome after MPs enters the soil, but little is known about the microbial utilization processes of DOM released by different types and aging of MPs incorporated into soil and more importantly, the underpinning mechanisms regulating the structure of associated acting microbial networks [11, 25, 45, 66, 67]. Here, we used molecular chemistry to demonstrate at the molecular level that the incorporation of different MPs led to an increase in molecular weight of DOM in agricultural soil (and its molecular composition tended to be high in carbon and oxygen species), and the microbiome results also matched this pattern. We went further with metabolomics to reveal the process of MPs-DOM utilization by associated microbiomes and the microbial network complexity during this process. The complexity of soil DOM under the PE-MPs treatment, a major component of agriculturally used plastic products, is much lower than that under the PLA-MPs treatment, where the polymers are biodegradable; the microbial utilization pathways of PLA–MPs–DOM are more complex than those of PE–MPs–DOM.

The results showed that the addition of PE-MPs and PLA-MPs at low concentrations could alter the diversity of soil microbiome, which was closely related to the degree of plastic aging and addition ratios [10, 66]. Previous studies have shown that the leaching amount of DOM from plastic polymers can reach 3% of the total polymer mass, indicating that the difference in the spectral mean of soil DOM before and after MP incorporation may be due to MP-DOM [46, 68]. More importantly, the MPs were covered by soil during the whole experiment, which prevents the potential photodegradation of released nonvolatile compounds by solar radiation and allows more DOM to remain in the soil and be further utilized by the microbiome [29]. The changes of soil DOM by MP incorporations at the molecular level showed that the molecular weight of soil DOM increased and the degree of oxidation increased with growing incubation time of the MP-incorporated soil, while the molecular composition of soil DOM after MP incorporations tended to be homogeneous and dominated by substances with high carbon and high oxygen contents [30]. The higher molecular weight and higher oxidation of DOM in soils treated with aged PE-MPs compared to unaged PE-MPs may be due to the fact that the MPs-DOM produced from UV-aged MPs contained more oxygenated structures [68]. Moreover, since the biodegradable PLA-MPs have a more easily hydrolyzed oxygen-containing molecular structure, their ability to release DOM and oxygen-containing structures are more prominent [69, 70]. Thus, the aging process of nonbiodegradable MPs amplifies the disturbance of soil DOM, while biodegradable PLA-MPs are causing more changes in the soil in this regard.

The incorporation of MPs reduced microbial abundance and diversity, while increasing the modularity of microbial incorporation networks, and thus increasing the complexity of microbial interactions. Moreover, the incorporation of MPs had a selective effect on soil microbial communities at the phylum and genus levels, an effect also reported in previous studies [11, 67, 71]. However, the second and third most abundant bacteria in soils with added MPs were potentially pathogenic *Pseudomonas* and *Elizabethkingia*, which are thought to contribute significantly to the variation of resistance genes; in terms of fungi, the increase in *Neocosmospora* and *Fusarium* due to the addition of MPs may increase the chances of soil plant diseases (Supplementary Figs S13 and S17) [72, 73]. But how microbiomes utilize MPs and their derivatives is not clearly addressed by previous studies. Most studies have concluded that MPs, as complex compounds with a predominantly carbon chain structure, are bound to alter soil carbon content or cycling when they enter the soil, thus adding additional carbon sources leading to microbial assimilation. However, the possibility of increasing the bioavailable carbon source by hydrolysis and fragmentation of molecular structure of PE-MPs and PLA-MPs in a short period of time is minimal, and the decomposition of MPs in soils takes place over decades [22, 74-76]. Therefore, could DOM production due to MPs themselves be the cause of perturbation of the soil microbiota? It has been shown that DOC leached from plastics stimulates microbial activities in the ocean, and that the combined action of MPs and MPs-DOM improves the metabolism of microbial communities [65, 77]. Therefore, it is reasonable to speculate that MPs-DOM can be utilized by microbiomes. To further reveal the possible microbial utilization pathways of MPs-DOM, we identified the potentially bioavailable compounds in MPs-DOM by Q-TOF and combined with untargeted metabolomics to identify the major MPs-DOM metabolic pathways, and the abundance of regulatory genes in the pathways also corresponded well with the substance in the metabolomic results. Moreover, the main bacterial and fungal species involved in the metabolism and the microbial species that contribute to community change after MPs incorporation into soil are essentially the same. This serves as evidence that likely only specific members of the soil microbiome can utilize MPs-DOM. Bacterial metabolism for PE–MPs–DOM and PLA–MPs–DOM was mainly concentrated in *Proteobacteria, Actinobacteriota*, and *Bacteroidota*. Among the fungi, the main participants in PE–MPs–DOM metabolism were *Penicillium* (*Ascomycota* phylum) and *Trichoderma* (*Ascomycota* phylum), whereas, in the PLA–MPs–DOM fungal metabolism, *Thelonectria* (*Ascomycota* phylum), *Fusarium* (*Ascomycota* phylum), and *Auricularia* (*Basidiomycota* phylum) played a major role. Bacteria likely play a major role in MPs-DOM metabolism compared to fungi, which may be because the key players in the rapid carbon cycling pathway in soils are usually bacteria, whereas fungi are mainly responsible for the decomposition of recalcitrant organic matter [78, 79].

Unlike natural DOM in a typical aquatic environment, MPs-DOM is mainly composed of low molecular weight fractions (<350 Da), which is consistent with the peak of soil DOM in the FT-ICP-MS spectra of aged and unaged PE-MPs-mixed soil at 100–350 Da (Supplementary Fig. S9) [15]. The fluorescence index values also indicate that soil DOM tended to originate from microbial activities in this study (Supplementary Fig. S7). Therefore, we can speculate that most of the changes that occurred in soil DOM after MP incorporation likely result from the microbial metabolism for MPs-DOM. We remain aware of the need to further track elemental transformations of MPs-DOM during soil metabolism through isotope labeling experiments and metagenomic data, as well as tangible evidence of microbiome contributions to this process. Nevertheless, our findings contribute to the conceptualization and possibilities of soil microbial involvement in the metabolism of MPs-DOM, which can be further developed in future studies.

### Environmental implications

It is estimated that by 2015, more than 6 billion tons of plastic waste had been generated, and in 2020 the annual plastic production reached 367 million tons, of which about 80% ended up in landfills or were discharged into the environment [80, 81]. Studies have suggested that the annual release of plastic waste into the terrestrial environment is likely 4–23 times that of the marine environment [23]. Therefore, it can be estimated that 235–281 million metric tons of plastic will enter the environment every year. The amount of plastic waste entering the soil is expected to increase by an order of magnitude by 2025 if development continues in an unabated fashion [82], and the release of MP-derived DOC would generate potentially significant impacts on microbial communities and carbon cycling in soil systems [65]. The values provided here are preliminary estimates only. As the plastic ages, it degrades and breaks down into smaller pieces. Biodegradable plastics, such as PLA, have been promoted as a more environmentally friendly alternative and may capture a larger market share in the future [83, 84]. The European Bioplastics and the Institutes for Bioplastics and Biocomposites in 2021 forecast the bioplastics market to be ~2.62 million tons by 2023 [85]. However, the ecological and environmental benefits of biodegradable plastics are still questionable. As with our results, biodegradation does not mean there is no impact on soil microbes and associated soil processes. On the contrary, compared with PE-MPs, the incorporation of PLA-MPs leads to a more rapid release of DOM, bringing more macromolecular organic matter to the environment, leading to differential changes in soil microbial communities, and disrupting the metabolic pathways of soil microbiomes to a greater extent. In addition to indirect inputs from the environment, plastic film mulching is currently the main pathway for plastic pollution in agriculture [86]. Our study showed that MPs significantly increased the accumulation of soil DOM fractions in soil and induced the development of soil DOM structures toward more aromatic and stable structures, improving the hazards posed by MPs-DOM and its sequestration in the soil. There was a greater degree of change following the addition of PLA-MPs than PE-MPs, which raises questions about the suitability of biodegradable plastics as an alternative in agriculture. As a hidden challenge posed by MPs to the agricultural environment, the disturbance of soil DOM by MPs-DOM and the further impact on agricultural output should receive extensive attention, in addition to the direct degradation of MPs in the soil.

## Conclusion

This study shows that soil DOM under the influence of MPs had higher molecular weight, aromaticity, and more abundant lignin-like and condensed aromatic components. MPs changed the abundance, community composition, and functional genes of soil microbiomes, which in turn plays an important role in the transformation of soil DOM. After identifying the microbially available components of MPs-DOM (PLA-MPs: terephthalate, acetate, oxalate, and L-lactate; PE–MPs–DOM: 4-nitrophenol, propanoate, and nitrate), we examined the microbial metabolic pathways of these components and found that PLA–MPs–DOM had more complex microbial metabolic pathways compared to PE–MPs–DOM. The bacteria involved in the transformation of MPs-DOM were concentrated in *Proteobacteria, Actinobacteriota,* and *Bacteroidota*, while the fungi were mainly *Ascomycota* and *Basidiomycota*. The overall contribution of bacteria in the metabolism of MPs-DOM was greater than that of fungi. Compared to PE-MPs, the incorporation of biodegradable PLA-MPs increases the oxygen content in soil DOM and severely reduces the microbial diversity of agricultural soil. This study reveals the effect of MPs incorporation on the molecular composition of soil DOM, elucidates the microbial metabolic pathways of MPs-DOM, and lays the foundation for further revealing the relationship between soil DOM and microbial community under the influence of MPs in agricultural production.

## Supplementary Material

Supplementary_wrad017

## Data Availability

Raw data for sequencing of bacteria and fungi were deposited in the NCBI BioProject database under the accession numbers PRJNA1021161 and PRJNA1021359, respectively.
