# Supplementary material for: Microbial metabolism influences microplastic perturbation of dissolved organic matter in agricultural soils
Source: ISME J. 2024 Jan 10;18(1):wrad017. doi: 10.1093/ismejo/wrad017 (PMC10811734; doi:10.1093/ismejo/wrad017)
Supplement: Supplementary_wrad017 [file supplementary_wrad017.zip › Figure.S14.pdf]

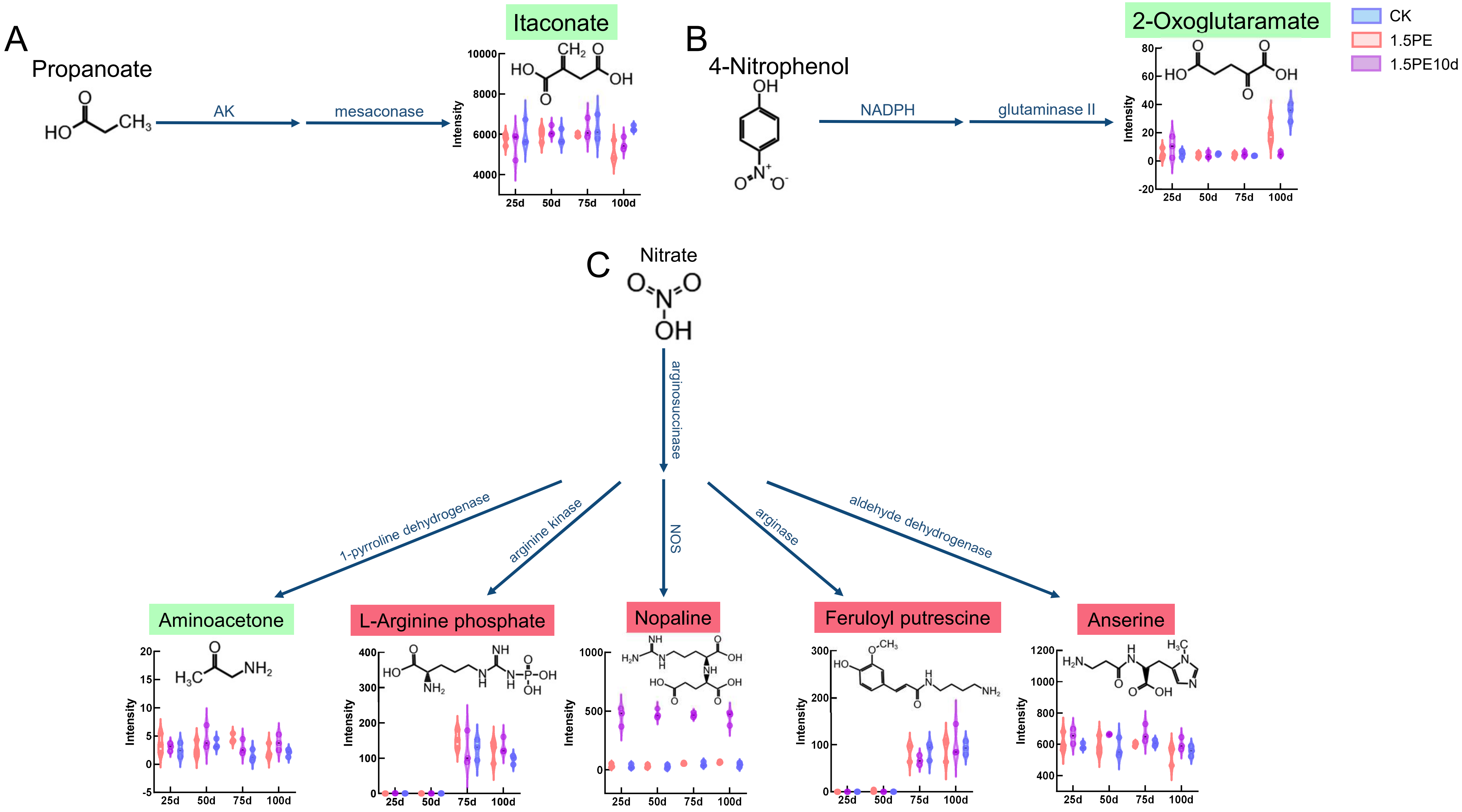

A
Propanoate
AK
mesaconase
Itaconate
B
4-Nitrophenol
NADPH
glutaminase II
2-Oxoglutaramate
CK
1.5PE
1.5PE10d
C
Nitrate
argininosuccinase
1-pyrroline dehydrogenase
arginine kinase
NOS
arginase
aldehyde dehydrogenase
Aminoacetone
L-Arginine phosphate
Nopaline
Feruloyl putrescine
Anserine
Intensity
25d
50d
75d
100d
