# Supplementary material for: Microbial metabolism influences microplastic perturbation of dissolved organic matter in agricultural soils
Source: ISME J. 2024 Jan 10;18(1):wrad017. doi: 10.1093/ismejo/wrad017 (PMC10811734; doi:10.1093/ismejo/wrad017)
Supplement: Supplementary_wrad017 [file supplementary_wrad017.zip › Figure.S3.pdf]

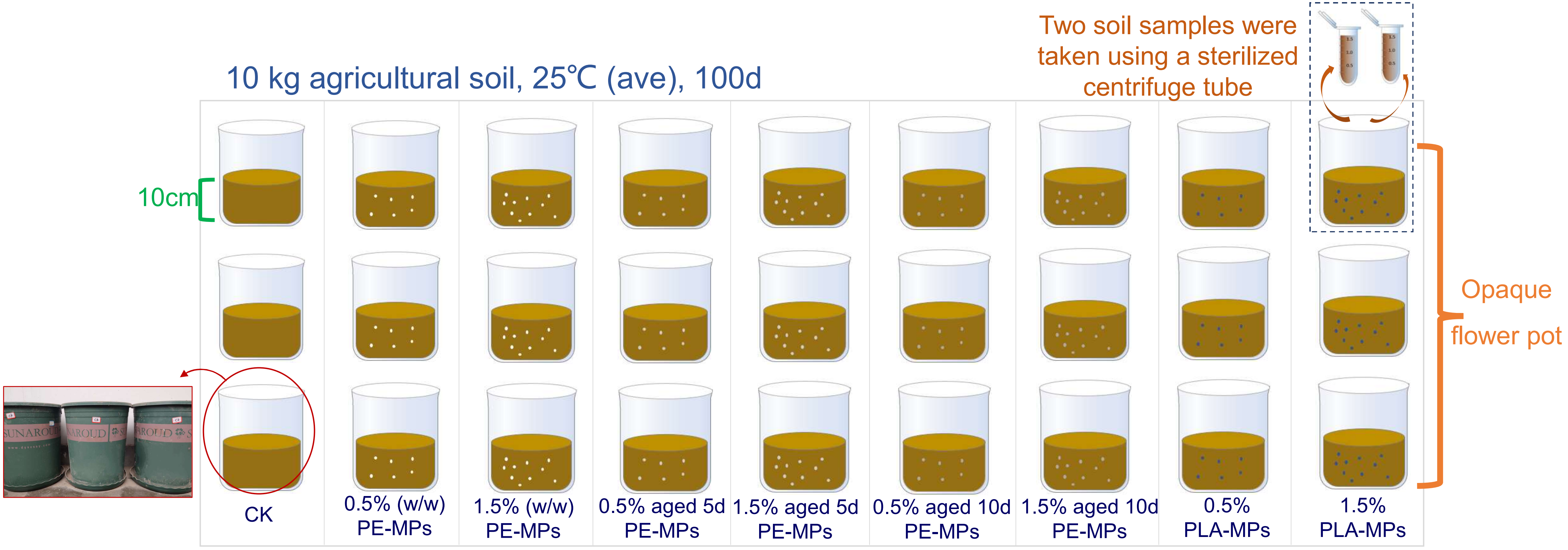
10 kg agricultural soil, 25℃ (ave), 100d
Two soil samples were taken using a sterilized centrifuge tube
10cm
Opaque flower pot
CK
0.5% (w/w) PE-MPs
1.5% (w/w) PE-MPs
0.5% aged 5d PE-MPs
1.5% aged 5d PE-MPs
0.5% aged 10d PE-MPs
1.5% aged 10d PE-MPs
0.5% PLA-MPs
1.5% PLA-MPs
