# Supplementary material for: Microbial metabolism influences microplastic perturbation of dissolved organic matter in agricultural soils
Source: ISME J. 2024 Jan 10;18(1):wrad017. doi: 10.1093/ismejo/wrad017 (PMC10811734; doi:10.1093/ismejo/wrad017)
Supplement: Supplementary_wrad017 [file supplementary_wrad017.zip › Supporting Information.pdf]

Supporting Information

Text S1. Basic information of experimental agricultural soil

The basic properties are as follows: pH 7.8 (2.5:1 water-soil ratio), 6.18 g/kg organic carbon content, 4.18 mg/kg available phosphorus, 0.81 g/kg total nitrogen (N) and 102.3 mg/kg available potassium. MPs content in original soil were (5.32 ± 0.2) × 10^{-2} items/kg.

Text S2. Extraction of DOM from soil

After air-dried in the dark for ~ 5 days, 10.0 g soil samples were accurately weighed into a 150 mL glass sample bottle and spiked with 100 mL ultrapure water to reach a water to soil ratio of 10:1 (V/w). The mixture was mixed and placed in a shaker (THZ-98C, Yiheng Scientific Instruments, China) at 200 rpm for 16 hours in the dark under 25 ± 2 °C [1, 2]. The suspensions were then centrifuged for 30 minutes at 4000 rpm (TDZ5-WS, Hunan Xiangyi Co., Ltd., China). Eventually, the supernatants were collected and filtered through 0.45μm glass fiber filter membrane to obtained soil DOM. The collected soil DOM was stored at 4 °C for further analysis.

Text S3. $SUVA_{254}$ calculation

Specific ultraviolet absorbances ($SUVA_{254}$) at 254 nm is an indicator that can reflect the content of humic substances, and indirectly reflects the aromaticity of DOM, the relative content of unsaturated double bonds, and the hydrophilicity and hydrophobicity of organic substances [3]. $SUVA_{254}$ is calculated as the ratio of UV_{254} (AU/cm) to DOC (mg/L) ($SUVA_{254} = (UV_{254}/DOC) \times 100$) in liters per milligram of carbon per meter (L/mg C · m).

Text S4. 3D-EEM analysis

3D-EEMs of extracted soil DOM were acquired using a fluorescence spectrophotometer (RF-6000, Shimadzu, Japan) with a 1 cm quartz cuvette. The excitation and emission scanning ranges were 250–600 nm at an increment of 5 nm and 200–650 nm at an increment of 5 nm, respectively. Excitation and emission slit widths were set to 5 nm, and the scan speed was 12000 nm min^{-1}. To avoid fluorescence inner-filtering, a criterion of an absorbance-based approach was employed.

Text S5. PARAFAC modeling

To decompose the fluorescence components in extracted soil DOM, PARAFAC modeling was performed using MATLAB 2014a (Mathworks, Natick, MA, USA) along with the DOM Fluor toolbox [4]. The PARAFAC was tested using 3-7 components models for the compost DOM dataset with the entire EEM dataset, and the split half analysis was used to verify the best allocated components after determining discrete value. Finally, the components separated by PARAFAC were determined according to the peak value and position. The maximum fluorescence intensities (F_{max}) (R.U.) were adopted to express the relative level of each component [5]. Based on 3D-EEM data to obtain biological index (BIX, revealing the biodegradation of DOM), humification index (HIX, positively correlated with DOM humification) and fluorescence index (FI, usually used to distinguish the origin of DOM: terrestrial organisms: $1.2 < FI < 1.5$, microbial activity: $1.7 < FI < 2.0$) [6-8].

Text S6. Characterization of MPs-DOM analysis

The relevant settings of quadrupole time-of-flight (Q-TOF) mass spectrometry are as follows:

Quality range (m/z): TOF part: m/z 100-10,000; Quadrupole part: m/z 50-4,000; Mass spectrum resolution: > 45,000 (M/Z:600-1000); Sensitivity: ESI MS/MS positive ion mode: S/N>1500:1; Scanning speed: MS/MS mode: 30 MS/MS spectrum/second; Quality accuracy: MS/MS mode: < 2 ppm; Positive and negative ion switching speed: 1.5 seconds; Detector acquisition speed: 4GHz.

Data acquisition mode: full SCAN; target ion mass spectrometry/mass spectrometry acquisition (target MS/MS); Automatic Mass Spectrometry/Mass Spectrometry Acquisition (auto MS/MS).

Qualitative analysis of data: able to process multiple sets of data at the same time; Molecular feature extraction function: extract compound peaks from mass data; Automatically calculate the chemical formula of each peak, calculate the isotope ratio; Compound finding function of MS/MS spectra with data correlation model; Mass accuracy, isotope ratio to determine the final formula.

Analyze: The qualitative analysis software Compoud discoverer of Thermo Field was used for qualitative processing, and the databases used were mzCloud, chemspider and mzvault.

Text S7. Purification of extracted soil DOM for FT-ICR-MS analysis

An established solid phase extraction (SPE) procedure was used for desalinization of the collected soil DOM, which was done by using a proprietary polymer Agilent Bond Elut PPL resin [9]. The detailed purification processes were as follows:

(1) Filtrate the collected soil DOM through 45 μm filter to remove particulates and acidified to pH 2-2.5 using HCl.

(2) Rinse the PPL (500 mg, 6 mL) cartridge with 1 volume of MeOH (HPLC grade).

(3) Rinse the PPL cartridge with 2 volumes of acidified ultrapure water (pH 2-2.5 with trace metal grade HCl).

(4) Pass the acidified sample through the PPL cartridge, ensuring that the flow rate was lower than 20 mL min^{-1}.

(5) Rinse the PPL cartridge with 2 volumes of acidified ultrapure water.

(6) Dry the PPL cartridge under a gentle stream of ultra-high purity N_2.

(7) Elute DOM from the PPL cartridge with 1-2 cartridge volumes of HPLC grade MeOH, by gravity ensuring that the flow rate was lower than 1 mL min^{-1}.

(8) Store extract in freezer until FT-ICR-MS analysis. The recovery of DOC was 67±6%.

Text S8. FT-ICR-MS Analysis of soil DOM

The FT-ICR-MS analysis of DOM was performed using a negative-ion Apollo II ESI coupled with a 15 T Bruker Apex Ultra FT-ICR mass spectrometer. The ion source is an electrospray ion source in negative ion mode[10]. The instrument was calibrated with 10 mmol/L sodium formate before sample detection, and the internal standard was calibrated with soluble organic matter (known molecular formula) after sample detection [9]. After calibration, the detected mass errors are all less than 1 ppm. The FT-ICR-MS analysis of DOM was performed using a negative-ion Apollo II ESI coupled with a 15 T Bruker Apex Ultra FT-ICR mass spectrometer. The soil DOM samples were diluted to about 100 mg L^{-1} and injected into the ESI source at a rate of 250 μL h^{-1}. The typical operating conditions for negative-ion ESI analysis were: 4.0 kV spray shield voltage, 4.5 kV capillary column introduced voltage, and -320 V capillary column end voltage. Ions were accumulated in the collision cell for 1.0 The soil DOM samples were diluted to about 100 mg L^{-1} and injected into the ESI source at a rate of 250 μL h^{-1}. The typical operating conditions for negative-ion ESI analysis were: 4.0 kV spray shield voltage, 4.5 kV capillary column introduced voltage, and -320

V capillary column end voltage. Ions were accumulated in the collision cell for 1.0 s then transferred into the ICR cell with a 1.1 ms time of flight. The ion transformation parameter for the quadrupole was optimized at m/z 200. The mass range was m/z 150-800. A total of 128 scans with 4M word size was accumulated to enhance the signal-to noise ratio. The mass spectrometer was calibrated with a known mass series in the sample. Once the exact masses of the detected ions have been determined, their molecular formulas were batch-calculated by an inhouse software tool (mass error, ≤0.1 ppm). Data analysis was performed using in house software. Briefly, data analysis was performed by selecting a two-mass scale expanded segment near the most abundant peak of the spectrum, followed by detailed identification of each peak. The peak of at least one of each class species was arbitrarily selected as a reference peak. Each class species and its isotopes with different double bond equivalents and carbon number values were searched within a tolerance of ±0.001 Kendrick mass defect (KMD).

Text S9. Calculations of Optical and Molecular Parameters of soil DOM based on FT-ICR-MS data

There were numerous optical and molecular proxies used in this study. The double bond equivalent (DBE) was calculated by 1+C-O-S-1/2(N+H). The modified aromatic index (AI) was calculated by DBE/(C-1/2×O-N-S). The nominal oxidation state of carbon (NOSC) was calculated by 4-((4C+H-3N+2O-2S)/C) in this study. The relative intensity was calculated by dividing each formula intensity by the sum of intensity of assigned formulas. Note: C, H, O, N, and S stand for the number of carbons, hydrogen, oxygen, nitrogen, and sulfur atoms in a particular molecular formula.

Test S10. Untargeted Metabolomics analysis

Metabolite extraction: First, weigh 50 mg of air-dried soil samples into 1 mL extractant ($V_{methanol}$: $V_{acetonitrile}$: V_{water}=2:2:1) containing 2 mg/L internal standard., and vortex for 30 s. Then, add porcelain beads into the mixed suspensions and grind for 10 min followed by sonication at 45 Hz (ice bath) for 10 min. The sonicated samples were allowed to stand at -20°C for one h and then centrifuged at 12000 rpm for 15 min at 4°C. Afterwards, 500 μL supernatant was carefully collected into an eppendorf tube. The collected extract was dried using a vacuum concentrator and redissolved in 160 μL extractant ($V_{acetonitrile}$: V_{water}=1:1). After vortex for 30 s and sonication in an ice water bath for 10 min, the mixed solutions were again centrifuged at 12000 rpm for 15 min at 4°C. Eventually, 120 μL of supernatant was carefully pipetted into 2 mL injection bottles, and 10 μL of each sample was mixed into quality control samples.

Detection: The extract was analyzed using a Waters Acquity I-Class PLUS UPLC tandem with a Waters Xevo G2-XS QTOF high-resolution mass spectrometer. The extract was separated using an Acquity UPLC HSS T3 column (2.1 × 100 mm, 1.8 μm). Both positive and negative ionization mode were used for mass spectra analysis. During positive ionization mode, the mobile phase was composed of 0.1% formic acid aqueous solution (A): 0.1% formic acid acetonitrile (B). Similar, 0.1% formic acid aqueous solution (A) 0.1% formic acid acetonitrile (B) was used for the negative ionization mode. The injection volumes were all 1μL. During the mass spectra detection, the MassLynx V4.2 software (Waters) was used to control the acquisition of the primary and secondary ions. In each data acquisition cycle, dual channel data acquisition is possible for both low and high collision energies. Electrospray ionization (ESI) mode was used to produce ions with capillary voltage of 2000 V (for positive ion mode) or -1500 V (for negative ion mode) and cone hole voltage

of 30 V. The ion source temperature is 150°C; the desolventizing gas temperature is 500°C. The flow rate of blowback gas is 50L/h; the flow rate of desolventizing gas is 800L/h.

Data processing: The raw data collected with MassLynx V4.2 were processed by Progenesis QI software for peak extraction, peak alignment, and other data processing operations. Based on the online METLIN database of Progenesis QI software and Bemec's own library for identification, the theoretical fragments were identified at the same time, and the mass number deviation was within 100 ppm.

Reference

1. Liu H, Yang X, Liu G, Liang C, Xue S, Chen H, *et al.* Response of soil dissolved organic matter to microplastic addition in Chinese loess soil. *Chemosphere.* 2017; 185:907-917.
2. Roth V-N, Lange M, Simon C, Hertkorn N, Bucher S, Goodall T, *et al.* Persistence of dissolved organic matter explained by molecular changes during its passage through soil. *Nat Geosci.* 2019; 12(9):755-+.
3. Tang SJ, Wang ZW, Zhi-Chao WU, Wang XH, Ji-Lai LU, Xia MFJZHKCES. Excitation-emission matrix fluorescence spectra analysis of dissolved organic matter in membrane bioreactor. 2009.
4. Zhu Y, Jin Y, Liu X, Miao T, Guan Q, Yang R, *et al.* Insight into interactions of heavy metals with livestock manure compost-derived dissolved organic matter using EEM-PARAFAC and 2D-FTIR-COS analyses. *J Hazard Mater.* 2021; 420:126532.
5. Stedmon CA, Bro R. Characterizing dissolved organic matter fluorescence with parallel factor analysis: a tutorial. *Limnol Oceanogr Meth.* 2008; 6:572-579.
6. Chen M, Huang Y, Wang C, Gao H. The conversion of organic nitrogen by functional bacteria determines the end-result of ammonia in compost. *Bioresour Technol.* 2020; 299:122599.
7. Leenheer JA, Wershaw RL, Brown GK, Reddy MM. Characterization and diagenesis of strong-acid carboxyl groups in humic substances. *Appl Geochem.* 2003; 18(3):471-482.
8. Mcknight DM, Boyer EW, Westerhoff PK, Doran PT, Kulbe T, Andersen DT. Spectrofluorometric characterization of dissolved organic matter for indication of precursor organic material and aromaticity. *Limnol Oceanogr.* 2001; 46(1):38-48.
9. Gonsior M, Powers LC, Williams E, Place A, Chen F, Ruf A, *et al.* The chemodiversity of algal dissolved organic matter from lysed Microcystis aeruginosa cells and its ability to form disinfection by-products during chlorination. *Water Res.* 2019; 155:300-309.
10. Xu W, Gao Q, He C, Shi Q, Hou Z-Q, Zhao H-Z. Using ESI FT-ICR MS to characterize dissolved organic matter in salt lakes with different salinity. *Environ Sci Technol.* 2020; 54(20):12929-12937.

Supplementary table legends

Table S1. Stoichiometric ranges of the classifications for van-Krevelen diagrams.

Table S2. Magnitude-weighted (indicated by w as a subscript) averaged characteristics of DOM fractions extracted from CK, 1.5PE, 1.5PE10D, and 1.5PLA in 100 days.

Table S3. Data from ternary plots of bacterial communities affected by different MPs. The data correspond to Fig.2 (B).

Table S4. Data from ternary plots of fungal communities affected by different MPs. The data correspond to Fig.2 (E).

Table S5. Topological properties of networks of the MPs-soil bacteria.

Table S6. Topological properties of networks of the MPs-soil fungi.

Table S7. Q-TOF analysis of the composition of MPs-DOM identified by KEGG comparison of the metabolome to the microbially available components.

Table S8. Relevant parameters of the microbial co-occurrence network involved in the metabolism of MPs-DOM.

Supplementary figure legends

Fig. S1 The percentages of the (A) shape and (B) type of background MPs in sampled soil; Micrographs of MPs extracted from sampled soil. (C, D, E) fibers, (F, G, H) films and (I, J, K) fragments.

Fig. S2 SEM image of PLA-MPs (A), PE-MPs (B), PE with UV aging for 5 days (C) and PE with UV aging for 10 days (D).

Fig. S3 Schematic diagram of the experimental setups during incubation of different MPs in agricultural soils.

Fig. S4 UV_{254} (A) and DOC (B) images of different treatments.

Fig. S5 3D-EEM images of soils doped with different MPs.

Fig. S6 PARAFAC model output showing three distinct fluorescence components (up) and corresponding excitation/emission loadings (down) for different MPs components.

Fig. S7 Changes in the fluorescence index of soil DOM after adding different MPs: A: biological index (BIX, revealing the biodegradation of DOM), B: fluorescence index (FI, usually used to distinguish the origin of DOM: terrestrial organisms: $1.2 < FI < 1.5$, microbial activity: $1.7 < FI < 2.0$), C: humification index (HIX, positively correlated with DOM humification).

Fig. S8 van Krevelen diagrams of soil DOM extracted from control (CK), soil supplemented with 1.5% PE (1.5PE), 1.5%PE aged for 10 days (1.5PE10d), and 1.5% PLA.

Fig. S9 Negative FT-ICR-MS spectra of soil with CK (A), 1.5PE (B), 1.5PE10d (C) and 1.5PLA (D) after 100 days of culture; pie charts indicate the relative abundances of the CHO, CHON, CHOS, and

CHONS compounds. The four pie charts show the percentages of the different formula groups in different samples by intensity.

Fig. S10 Relative intensities of molecular formulas containing different numbers of oxygen atoms.

Fig. S11 Changes in soil AI, DBE, and NOSC index with the carbon number of DOM molecular formula after MPs incorporation.

Fig. S12 Shannon index (A: bacteria, B: Fungi) and venn diagram (C) of soil microbial community after MPs treatment based on OTU results.

Fig. S13 Mean relative abundance of bacteria (A) and fungi (B) at the genus level for different MPs.

Fig. S14 Microbial metabolic pathways of PEMPs-DOM upon entry into the soil. The bioavailable fraction in PEMPs-DOM was mainly propanoate (A), 4-nitrophenol (B), and nitrate (C). The red molecular formula indicates up-regulation of metabolites, and the green molecular formula indicates down-regulation of metabolites. Violin plots represent the abundance of microbial metabolic MPs-DOM products. The CK treatment is shown in blue, the 1.5PE treatment in orange, and the 1.5PE10d treatment by purple. The abundance of dominant genes in metabolic pathways is shown in Fig.S16. Error bars represent one standard deviation, n=3.

Fig. S15 Abundance of dominant genes in microbial metabolic pathways after PLAMPs-DOM entry into soil, including those of L-lactate (A), terephthalate (B), acetate (C), and oxalate (D). The CK treatment is shown in blue, and the 1.5PLA treatment by yellow. Error bars represent one standard deviation, n=3.

Fig. S16 Abundance of dominant genes in microbial metabolic pathways after PEMPs-DOM entry into soil, including those of propanoate (A), 4-nitrophenol (B), and nitrate (C). The CK treatment is shown in blue, the 1.5PE treatment in orange, and the 1.5PE10d treatment by purple. Error bars

represent one standard deviation, n=3.

Fig. S17 Normalized abundance of major microbiome members involved in MPs-DOM metabolism at the genus level, including bacterial abundance (A) and fungal abundance (C) in the PEMPs-treated group and bacterial abundance (B) and fungal abundance (D) in the PLAMPs-treated group. Normalized abundances were obtained by LOG transformation of bacterial and fungal abundances at the genus level. By ranking the abundance of the microbiome with the related metabolic function genes, the microbiome (at the genus level) with the top ten abundances was the dominant microbiome.

Fig. S18 The proportion of the main microbiomes (A: bacterial, B: fungal, phylum level) involved in the utilization process of PEMPs-DOM and PLAMPs-DOM.

Fig. S19 Random forest analysis of microbiomes involved in the metabolism of PEMPs-DOM and PLAMPs-DOM. Screening of the top ten microbiome (genus level) involved in the production of each of the major metabolites for random forest analysis. Blue is the major metabolizing microbiomes of PLAMPs-DOM and red is the major metabolizing microbiomes of PEMPs-DOM.

Fig. S20 Overall contribution of bacteria and fungi in the metabolism of MPs-DOM based on the results of random forest analysis.

Fig. S21 Mean values of nodes in the co-occurrence network of microbiomes (A: bacterial, B: fungal, phylum level) involved in the metabolism of MPs-DOM.
