# Supplementary material for: Microbial metabolism influences microplastic perturbation of dissolved organic matter in agricultural soils
Source: ISME J. 2024 Jan 10;18(1):wrad017. doi: 10.1093/ismejo/wrad017 (PMC10811734; doi:10.1093/ismejo/wrad017)
Supplement: Supplementary_wrad017 [file supplementary_wrad017.zip › Table.S1.docx]

| Classes | H/C | O/C |
| --- | --- | --- |
| Lipids | 1.5<H/C≤2.0 | 0≤O/C≤0.3 |
| Protein | 1.5<H/C≤2.2 | 0.3<O/C≤0.67 |
| Carbohydrates | 1.5<H/C≤2.4 | 0.67≤O/C<1.2 |
| Unsaturated hydrocarbons | 0.7<H/C≤1.5 | O/C<0.1 |
| Lignins/cram-like | 0.7<H/C≤1.5 | 0.1<O/C<0.67 |
| Tannins | 0.6≤H/C≤1.5 | 0.67<O/C<1.2 |
| Aromatic structures | 0.2<H/C≤0.7 | O/C≤0.67 |
