# Supplementary material for: Microbial metabolism influences microplastic perturbation of dissolved organic matter in agricultural soils
Source: ISME J. 2024 Jan 10;18(1):wrad017. doi: 10.1093/ismejo/wrad017 (PMC10811734; doi:10.1093/ismejo/wrad017)
Supplement: Supplementary_wrad017 [file supplementary_wrad017.zip › Table.S2.docx]

| Treatments | C_w_ | H_w_ | O_w_ | MW_w_ | H/C_w_ | O/C_w_ | DBE_w_ | AI_w_ | NOSC_w_ |
| --- | --- | --- | --- | --- | --- | --- | --- | --- | --- |
| CK | 20.53 | 29.15 | 7.81 | 409.98 | 1.50 | 0.39 | 6.57 | 0.11 | -0.65 |
| 1.5PE | 21.12 | 28.24 | 7.49 | 414.87 | 1.37 | 0.39 | 7.95 | 0.20 | -0.50 |
| 1.5PE10D | 22.48 | 27.54 | 8.50 | 439.73 | 1.32 | 0.38 | 8.95 | 0.23 | -0.56 |
| 1.5PLA | 23.65 | 27.43 | 11.41 | 504.73 | 1.24 | 0.51 | 10.52 | 0.35 | -0.52 |
