# Supplementary material for: Microbial metabolism influences microplastic perturbation of dissolved organic matter in agricultural soils
Source: ISME J. 2024 Jan 10;18(1):wrad017. doi: 10.1093/ismejo/wrad017 (PMC10811734; doi:10.1093/ismejo/wrad017)
Supplement: Supplementary_wrad017 [file supplementary_wrad017.zip › Table.S3.docx]

| Time | Group | Bacteria (phylum level) | | | | |
| --- | --- | --- | --- | --- | --- | --- |
|  |  | *Actinobacteria* | *Proteobacteria* | *Bacteroidetes* | *Acidobacteria* | *Firmicutes* |
| 25d | CK | 0.2756 | 0.3022 | 0.3306 | 0.2299 | 0.4266 |
|  | 1.5PE | 0.3102 | 0.3315 | 0.2828 | 0.4688 | 0.2663 |
|  | 1.5PLA | 0.4142 | 0.3663 | 0.3866 | 0.3013 | 0.3071 |
|  | 1.5PE | 0.3804 | 0.2887 | 0.2588 | 0.3334 | 0.4247 |
|  | 1.5PE5d | 0.2549 | 0.4506 | 0.3685 | 0.4078 | 0.1212 |
|  | 1.5PE10d | 0.3648 | 0.2607 | 0.3727 | 0.2588 | 0.4541 |
| 50d | CK | 0.3305 | 0.2952 | 0.3626 | 0.4318 | 0.3119 |
|  | 1.5PE | 0.2736 | 0.3400 | 0.3821 | 0.3008 | 0.3067 |
|  | 1.5PLA | 0.3959 | 0.3647 | 0.2553 | 0.2674 | 0.3814 |
|  | 1.5PE | 0.2931 | 0.3502 | 0.3294 | 0.3119 | 0.3156 |
|  | 1.5PE5d | 0.3146 | 0.3097 | 0.3592 | 0.3313 | 0.3506 |
|  | 1.5PE10d | 0.3923 | 0.3401 | 0.3115 | 0.3568 | 0.3338 |
| 75d | CK | 0.3038 | 0.3344 | 0.3242 | 0.3638 | 0.3448 |
|  | 1.5PE | 0.4533 | 0.3774 | 0.2406 | 0.3108 | 0.2782 |
|  | 1.5PLA | 0.2430 | 0.2882 | 0.4352 | 0.3254 | 0.3770 |
|  | 1.5PE | 0.4117 | 0.3513 | 0.2603 | 0.2797 | 0.3179 |
|  | 1.5PE5d | 0.2874 | 0.3296 | 0.3395 | 0.3757 | 0.3355 |
|  | 1.5PE10d | 0.3009 | 0.3191 | 0.4002 | 0.3446 | 0.3466 |
| 100d | CK | 0.2934 | 0.3483 | 0.3252 | 0.4069 | 0.2917 |
|  | 1.5PE | 0.2470 | 0.3331 | 0.4192 | 0.2697 | 0.3297 |
|  | 1.5PLA | 0.4595 | 0.3187 | 0.2556 | 0.3234 | 0.3786 |
|  | 1.5PE | 0.3049 | 0.3417 | 0.3263 | 0.3013 | 0.3347 |
|  | 1.5PE5d | 0.3329 | 0.3410 | 0.3238 | 0.4012 | 0.3057 |
|  | 1.5PE10d | 0.3622 | 0.3173 | 0.3498 | 0.2976 | 0.3595 |
