# Supplementary material for: Microbial metabolism influences microplastic perturbation of dissolved organic matter in agricultural soils
Source: ISME J. 2024 Jan 10;18(1):wrad017. doi: 10.1093/ismejo/wrad017 (PMC10811734; doi:10.1093/ismejo/wrad017)
Supplement: Supplementary_wrad017 [file supplementary_wrad017.zip › Table.S4.docx]

| Time | Group | Fungi (phylum level) | | | | |  |
| --- | --- | --- | --- | --- | --- | --- | --- |
|  |  | *Aphelidiomycota* | *Ascomycota* | *Basidiomycota* | *Chytridiomycota* | *Mortierellomycota* | *Rozellomycota* |
| 25d | CK | 1.0000 | 0.2553 | 0.4135 | 0.9625 | 0.6056 | 0.0000 |
|  | 1.5PE | 0.0000 | 0.3287 | 0.4685 | 0.0001 | 0.2796 | 0.0000 |
|  | 1.5PLA | 0.0000 | 0.4160 | 0.1179 | 0.0375 | 0.1148 | 1.0000 |
|  | CK | 1.0000 | 0.2700 | 0.3350 | 0.6775 | 0.4568 | 0.0000 |
|  | 1.5PE | 0.0000 | 0.2902 | 0.5695 | 0.2961 | 0.4566 | 0.0000 |
|  | 1.5PE10d | 0.0000 | 0.4398 | 0.0955 | 0.0264 | 0.0866 | 1.0000 |
| 50d | CK | 0.0000 | 0.2920 | 0.4933 | 0.0000 | 0.5828 | 0.0014 |
|  | 1.5PE | 1.0000 | 0.2841 | 0.3960 | 0.9610 | 0.3913 | 0.9986 |
|  | 1.5PLA | 0.0000 | 0.4239 | 0.1107 | 0.0390 | 0.0259 | 0.0000 |
|  | CK | 0.0000 | 0.3315 | 0.4439 | 0.0000 | 0.4146 | 0.0007 |
|  | 1.5PE | 0.0863 | 0.3225 | 0.3564 | 0.9459 | 0.2784 | 0.5237 |
|  | 1.5PE10d | 0.9137 | 0.3460 | 0.1998 | 0.0541 | 0.3069 | 0.4756 |
| 75d | CK | 0.3626 | 0.2510 | 0.5626 | 0.8138 | 0.4190 | 0.0000 |
|  | 1.5PE | 0.6374 | 0.2992 | 0.3945 | 0.0000 | 0.5309 | 1.0000 |
|  | 1.5PLA | 0.0000 | 0.4498 | 0.0429 | 0.1862 | 0.0500 | 0.0000 |
|  | CK | 0.3626 | 0.2876 | 0.4221 | 0.5146 | 0.3401 | 0.0000 |
|  | 1.5PE | 0.6374 | 0.3428 | 0.2960 | 0.0000 | 0.4308 | 0.0000 |
|  | 1.5PE10d | 0.0000 | 0.3696 | 0.2819 | 0.4854 | 0.2291 | 1.0000 |
| 100d | CK | 1.0000 | 0.2662 | 0.5475 | 0.0229 | 0.3758 | 0.9333 |
|  | 1.5PE | 0.0000 | 0.3247 | 0.3661 | 0.4134 | 0.2685 | 0.0667 |
|  | 1.5PLA | 0.0000 | 0.4091 | 0.0864 | 0.5637 | 0.3557 | 0.0000 |
|  | CK | 0.0113 | 0.2812 | 0.4828 | 0.0525 | 0.2496 | 0.7436 |
|  | 1.5PE | 0.0000 | 0.3430 | 0.3228 | 0.9475 | 0.1783 | 0.0532 |
|  | 1.5PE10d | 0.9887 | 0.3759 | 0.1944 | 0.0000 | 0.5721 | 0.2033 |
