# Supplementary material for: Microbial metabolism influences microplastic perturbation of dissolved organic matter in agricultural soils
Source: ISME J. 2024 Jan 10;18(1):wrad017. doi: 10.1093/ismejo/wrad017 (PMC10811734; doi:10.1093/ismejo/wrad017)
Supplement: Supplementary_wrad017 [file supplementary_wrad017.zip › Table.S5.docx]

|  | Number of nodes | Number of edges | Average degree | Average clustering coefficient | Average path length | Modularity |
| --- | --- | --- | --- | --- | --- | --- |
| CK | 543 | 609 | 1.122 | 0.16 | 1.294 | 0.948 |
| 0.5PE | 567 | 788 | 0.695 | 0.06 | 1.193 | 0.877 |
| 1.5PE | 553 | 650 | 1.422 | 0.126 | 1.697 | 0.752 |
| 0.5PEA5d | 457 | 650 | 1.422 | 0.126 | 1.697 | 0.755 |
| 0.5PEA10d | 556 | 352 | 0.931 | 0.115 | 1.186 | 0.952 |
| 1.5PEA5d | 387 | 352 | 1.535 | 0.126 | 2.099 | 0.91 |
| 1.5PEA10d | 615 | 605 | 0.984 | 0.081 | 1.547 | 0.945 |
| 0.5PLA | 1782 | 1622 | 14.709 | 0.212 | 4.347 | 0.586 |
| 1.5PLA | 1819 | 1721 | 12.491 | 0.207 | 4.409 | 0.649 |
