# Supplementary material for: Microbial metabolism influences microplastic perturbation of dissolved organic matter in agricultural soils
Source: ISME J. 2024 Jan 10;18(1):wrad017. doi: 10.1093/ismejo/wrad017 (PMC10811734; doi:10.1093/ismejo/wrad017)
Supplement: Supplementary_wrad017 [file supplementary_wrad017.zip › Table.S6.docx]

|  | Number of nodes | Number of edges | Average degree | Average clustering coefficient | Average path length | Modularity |
| --- | --- | --- | --- | --- | --- | --- |
| CK | 176 | 1393 | 15.830 | 0.988 | 1.000 | 3.187 |
| 1.5PE | 170 | 879 | 10.341 | 0.955 | 1.144 | 0.752 |
| 1.5PE10d | 171 | 1284 | 15.018 | 1 | 0.999 | 0.945 |
| 1.5PLA | 145 | 1050 | 14.483 | 0.951 | 1.024 | 0.649 |
