# Supplementary material for: Microbial metabolism influences microplastic perturbation of dissolved organic matter in agricultural soils
Source: ISME J. 2024 Jan 10;18(1):wrad017. doi: 10.1093/ismejo/wrad017 (PMC10811734; doi:10.1093/ismejo/wrad017)
Supplement: Supplementary_wrad017 [file supplementary_wrad017.zip › Table.S7.docx]

| MPs | Compound | Structure | Molecular formula | Molecular mass |
| --- | --- | --- | --- | --- |
| PLA | Terephthalate |  | C_8_H_6_O_4_ | 166.03 |
|  | Acetate |  | C_2_H_4_O_2_ | 60.05 |
|  | Oxalate |  | C_2_H_2_O_4_ | 90.03 |
|  | L-Lactate | 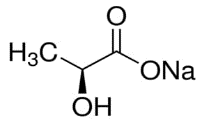 | C_3_H_6_O_3_ | 89.07 |
| PE | 4-Nitrophenol |  | C_6_H_5_NO_3_ | 139.11 |
|  | Nitrate |  | HNO_3_ | 62.01 |
|  | Propanoate |  | C_3_H_6_O_2_ | 74.07 |
