# Supplementary material for: Microbial metabolism influences microplastic perturbation of dissolved organic matter in agricultural soils
Source: ISME J. 2024 Jan 10;18(1):wrad017. doi: 10.1093/ismejo/wrad017 (PMC10811734; doi:10.1093/ismejo/wrad017)
Supplement: Supplementary_wrad017 [file supplementary_wrad017.zip › Table.S8.docx]

|  | PE-Bacteria | PLA-Bacteria | PE-Fungi | PLA-Fungi |
| --- | --- | --- | --- | --- |
| Number of edges | 2024 | 1897 | 25 | 325 |
| Number of positive edges | 1401 | 1411 | 25 | 323 |
| Number of negative edges | 623 | 486 | 0 | 2 |
| Number of nodes | 194 | 196 | 23 | 75 |
| Average degree | 20.866 | 19.357 | 2.174 | 8.667 |
| Network diameter | 3.591 | 4.065 | 4.445 | 4.754 |
| Average path length | 1.544 | 1.472 | 1.772 | 1.722 |
| Density | 3.591 | 4.065 | 0.099 | 0.117 |
| Relative modularity | 2.211 | 2.087 | 0.398 | 0.336 |
| Average clustering coefficient | 0.588 | 0.527 | 0.641 | 0.551 |
| Centralization degree | 0.187 | 0.162 | 0.083 | 0.221 |
| Centralization betweenness | 0.056 | 0.033 | 0.182 | 0.167 |
| Centralization closeness | 1.269 | 0.166 | 0.849 | 1.292 |
