# Supplementary figures and images for: Microbial metabolism influences microplastic perturbation of dissolved organic matter in agricultural soils
Source: ISME J. 2024 Jan 10;18(1):wrad017. doi: 10.1093/ismejo/wrad017 (PMC10811734; doi:10.1093/ismejo/wrad017)

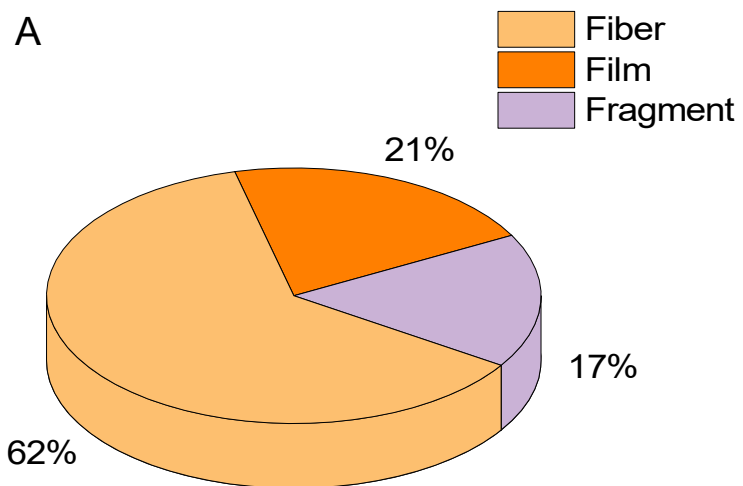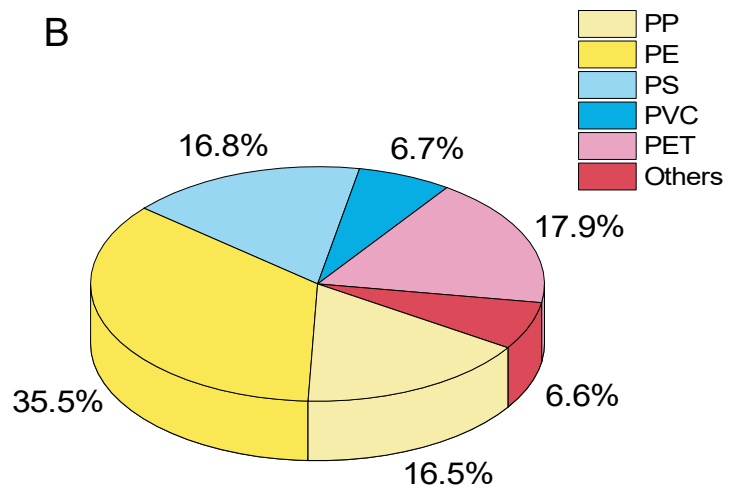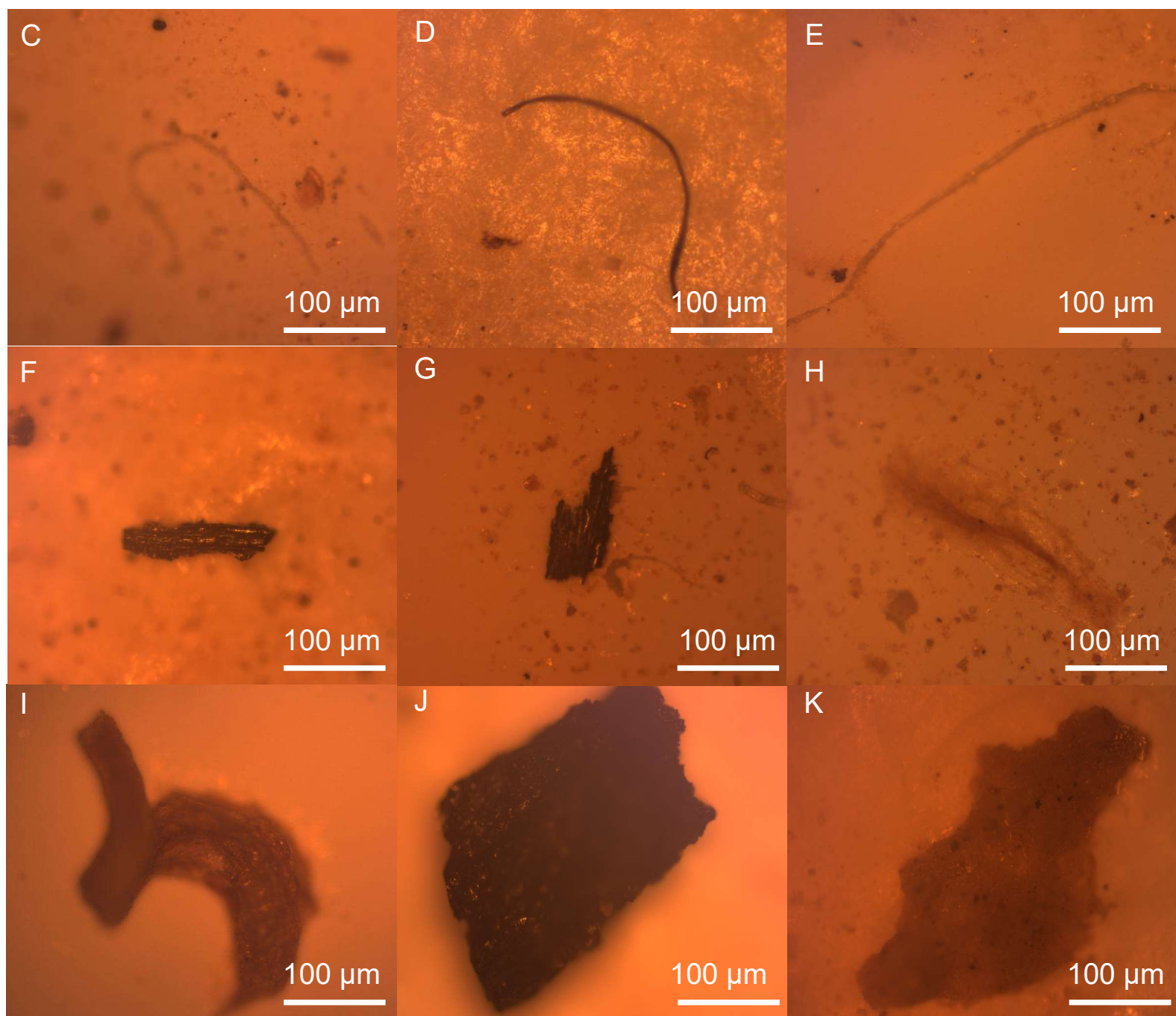

Supplement: Supplementary_wrad017 [file supplementary_wrad017.zip › Figure.S1.pdf]

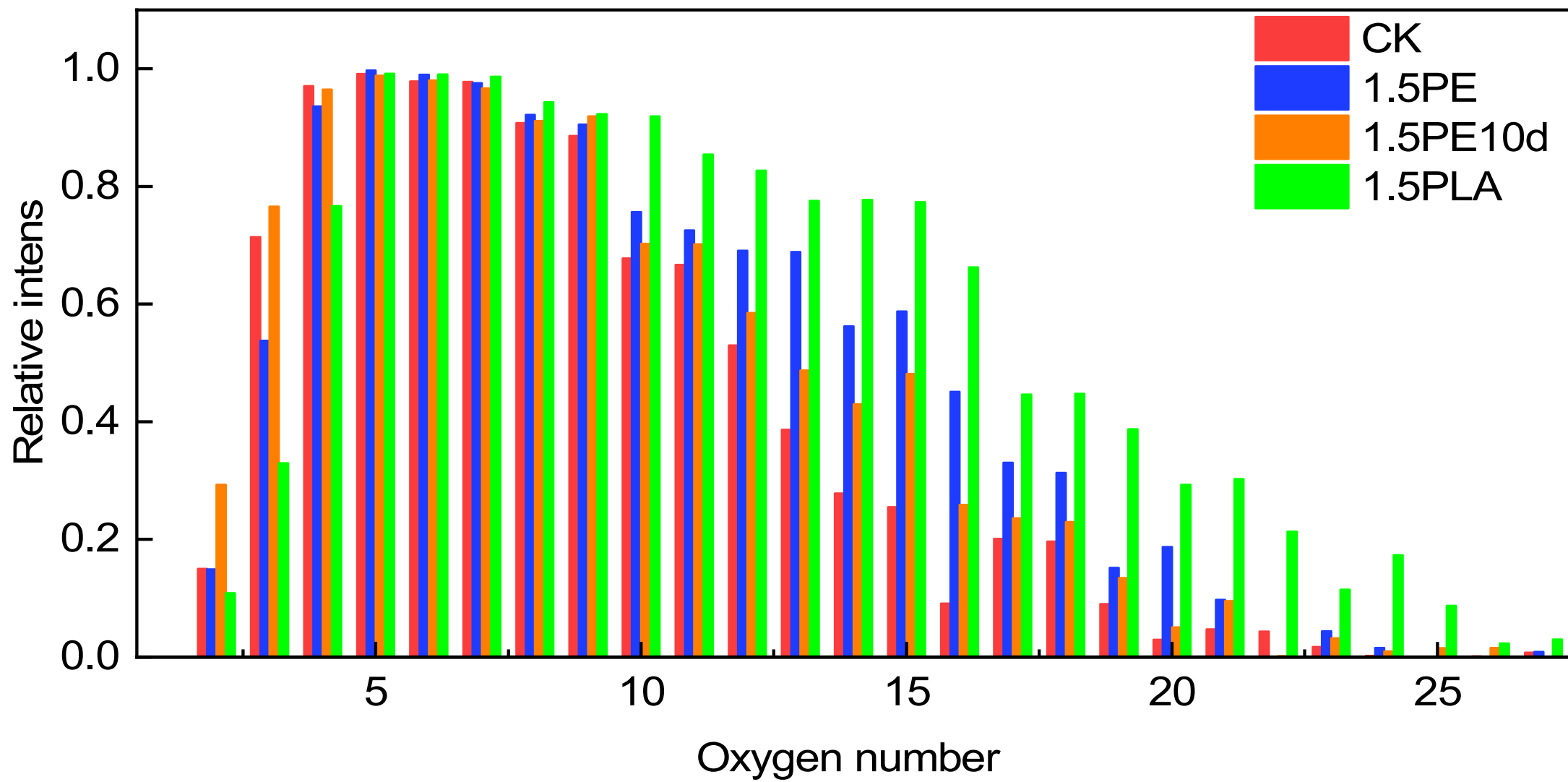

Supplement: Supplementary_wrad017 [file supplementary_wrad017.zip › Figure.S10.pdf]

CK

1.5PE

1.5PE10d

1.5PLA

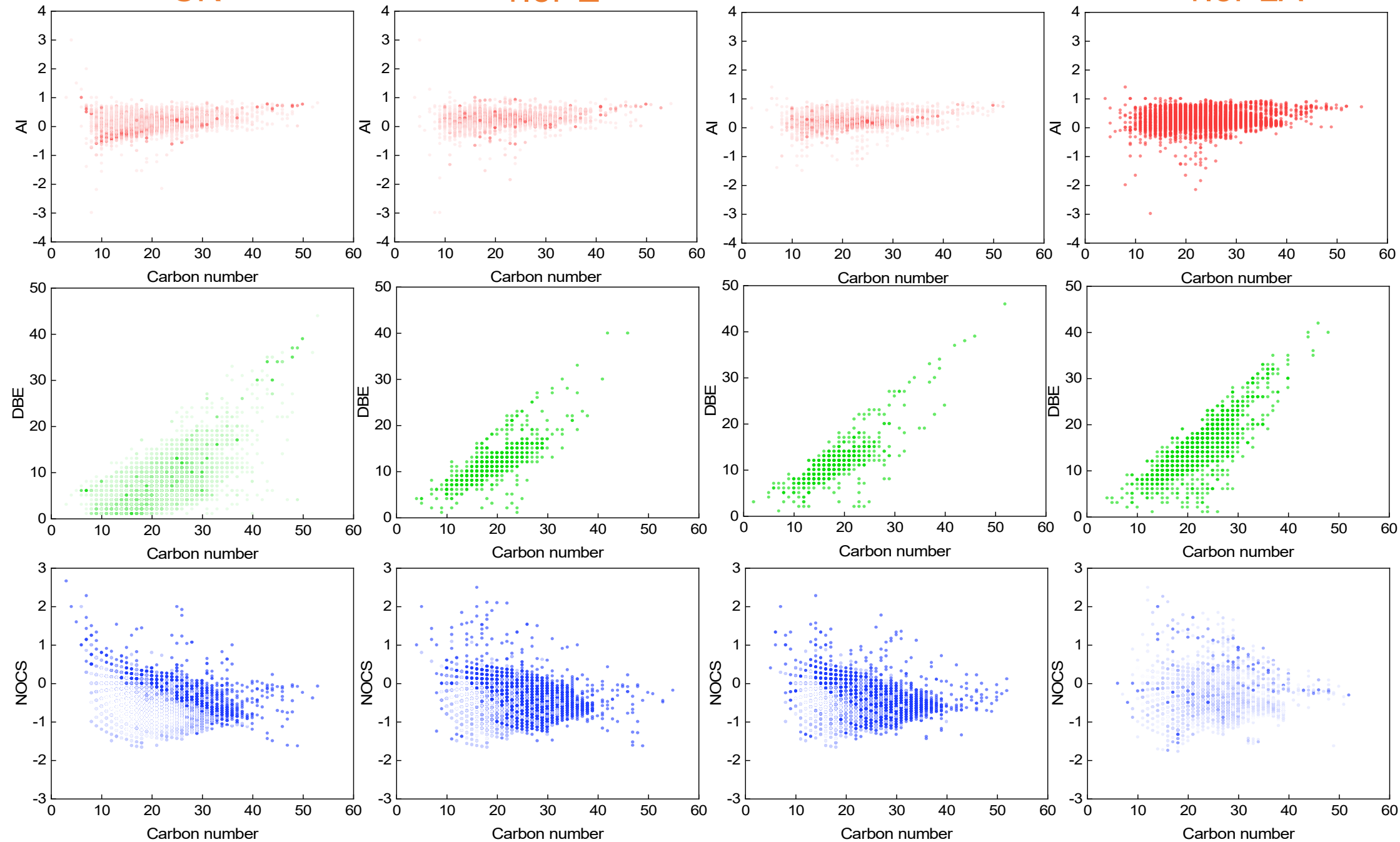

Supplement: Supplementary_wrad017 [file supplementary_wrad017.zip › Figure.S11.pdf]

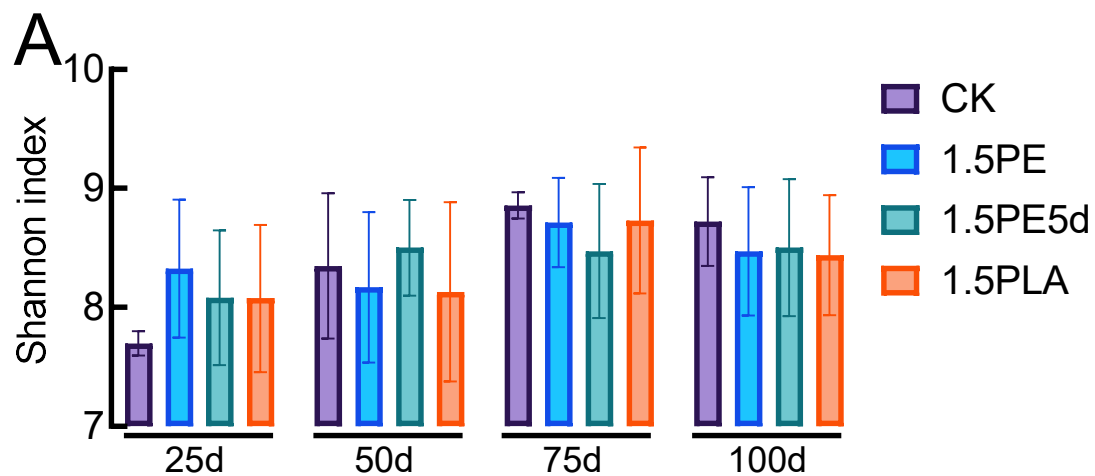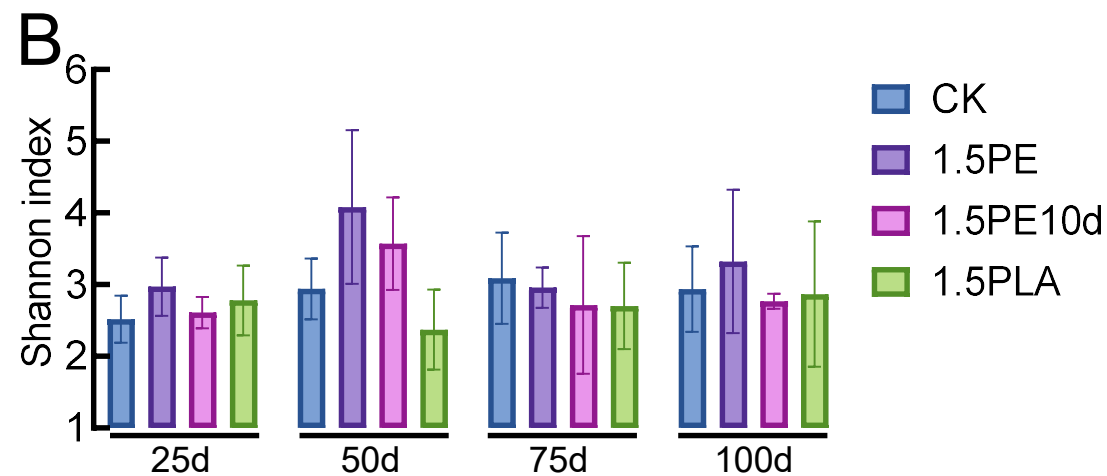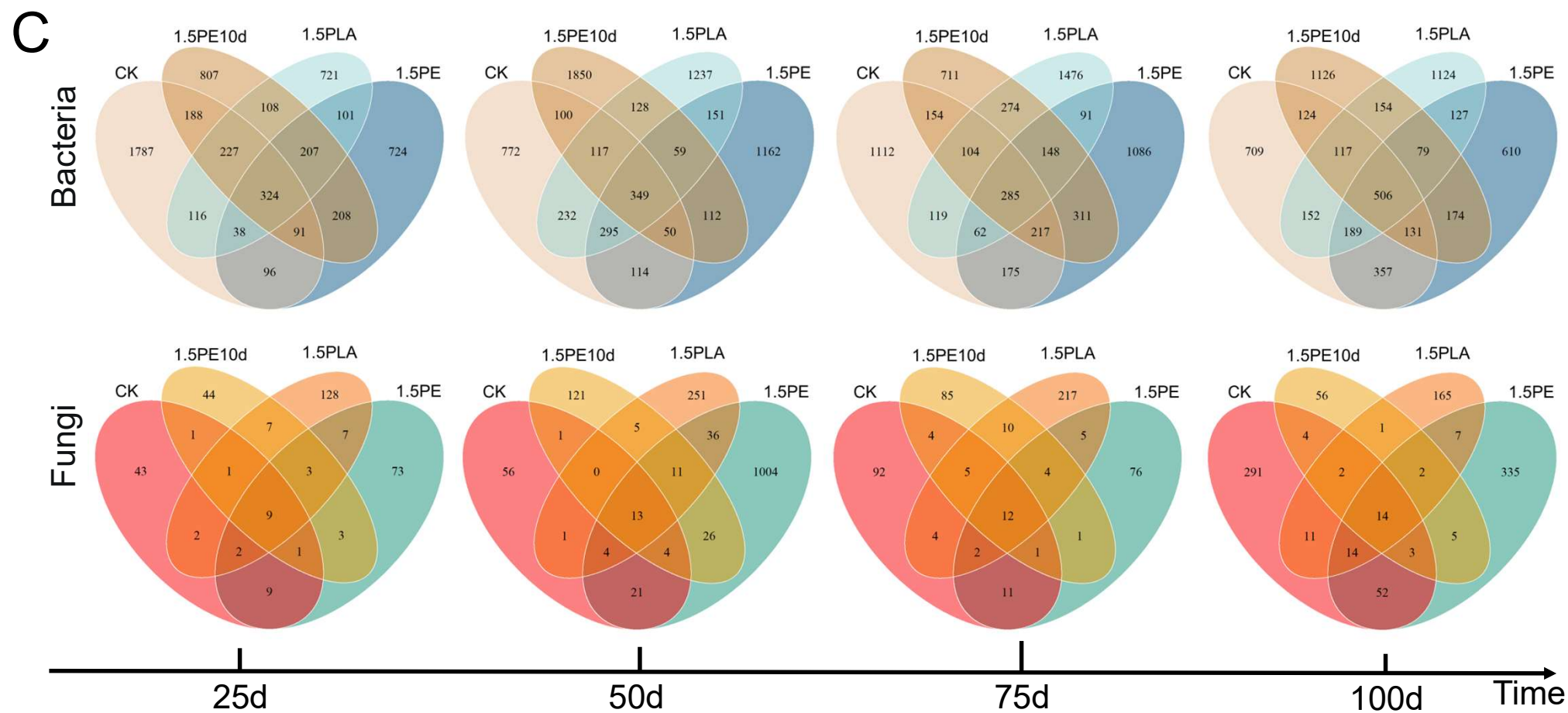

Supplement: Supplementary_wrad017 [file supplementary_wrad017.zip › Figure.S12.pdf]

A

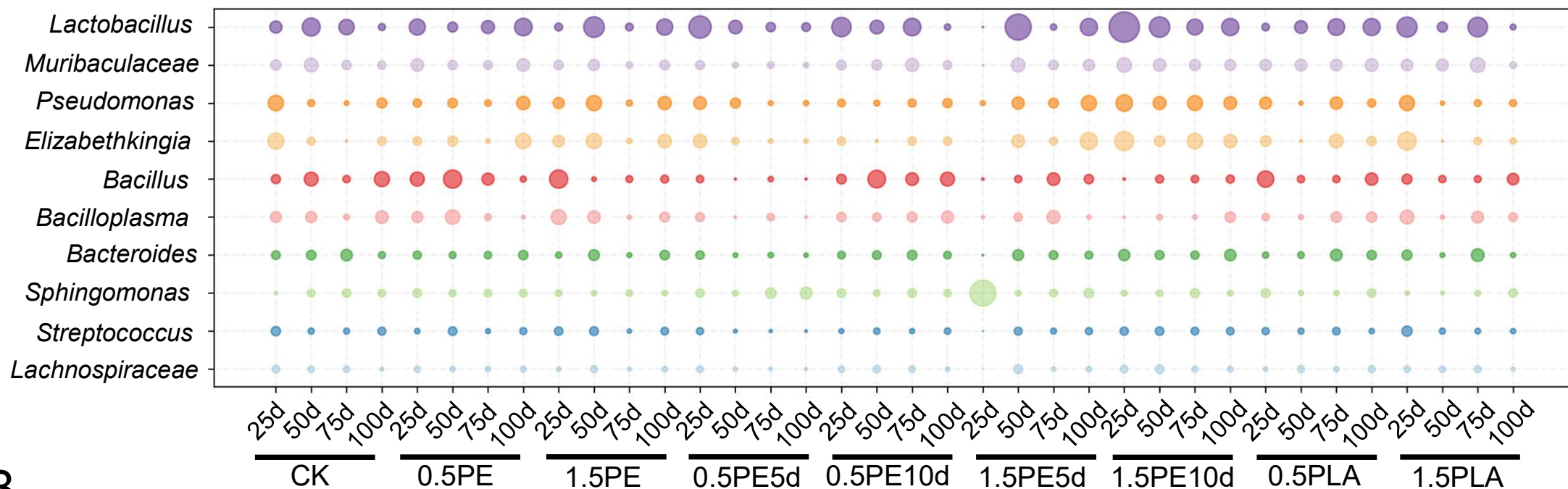

B

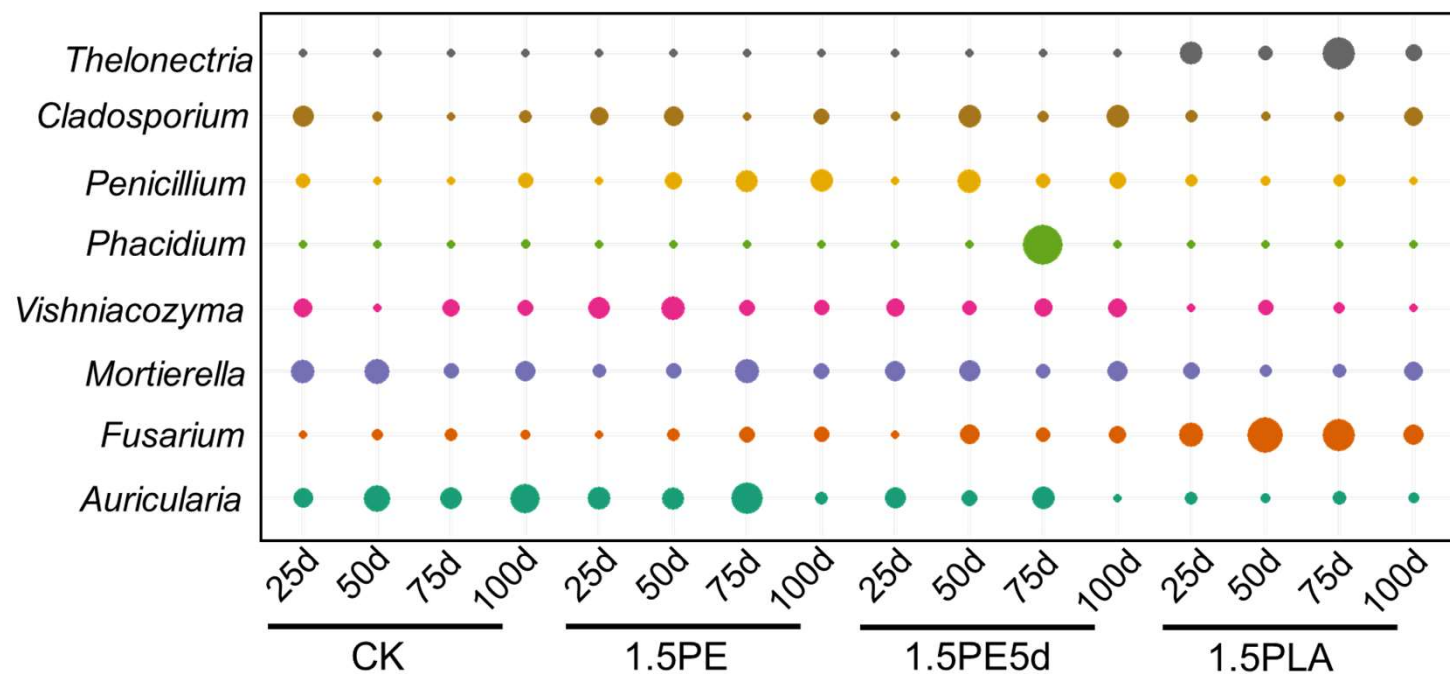

Supplement: Supplementary_wrad017 [file supplementary_wrad017.zip › Figure.S13.pdf]

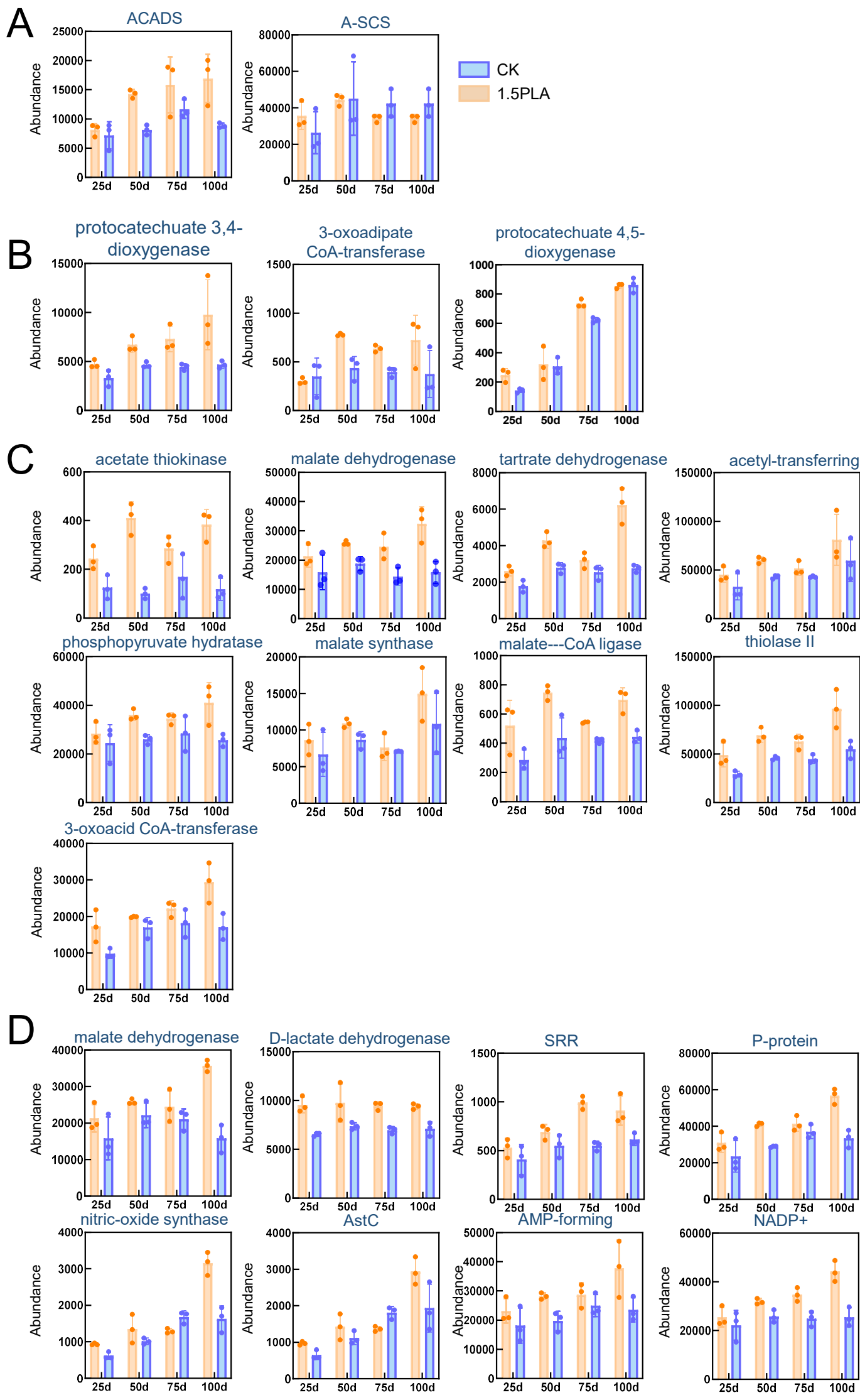

Supplement: Supplementary_wrad017 [file supplementary_wrad017.zip › Figure.S15.pdf]

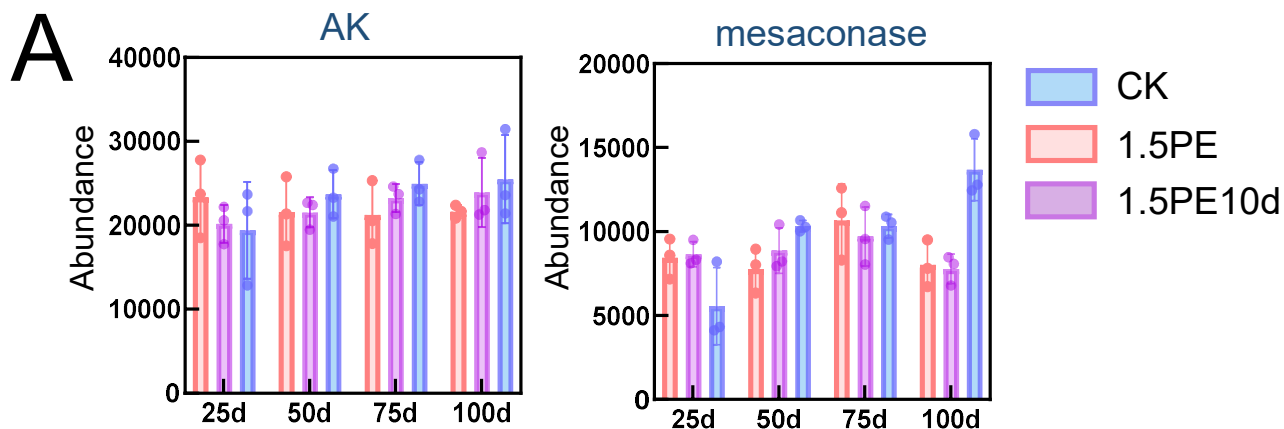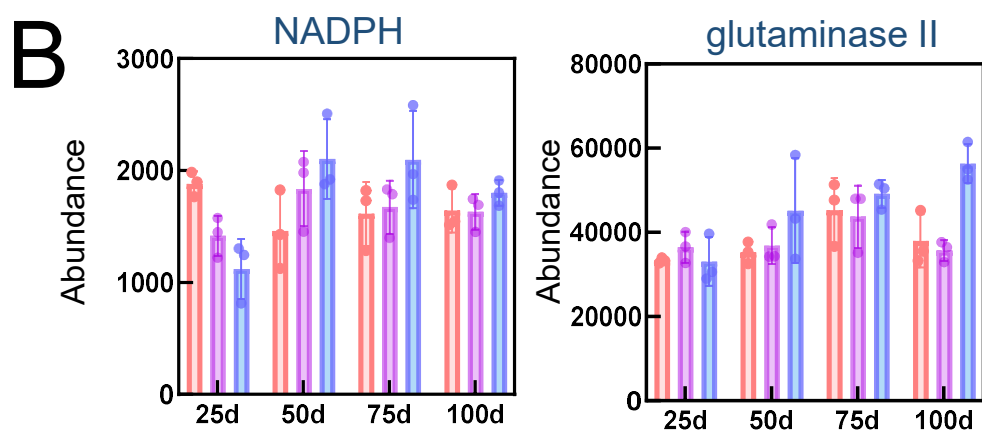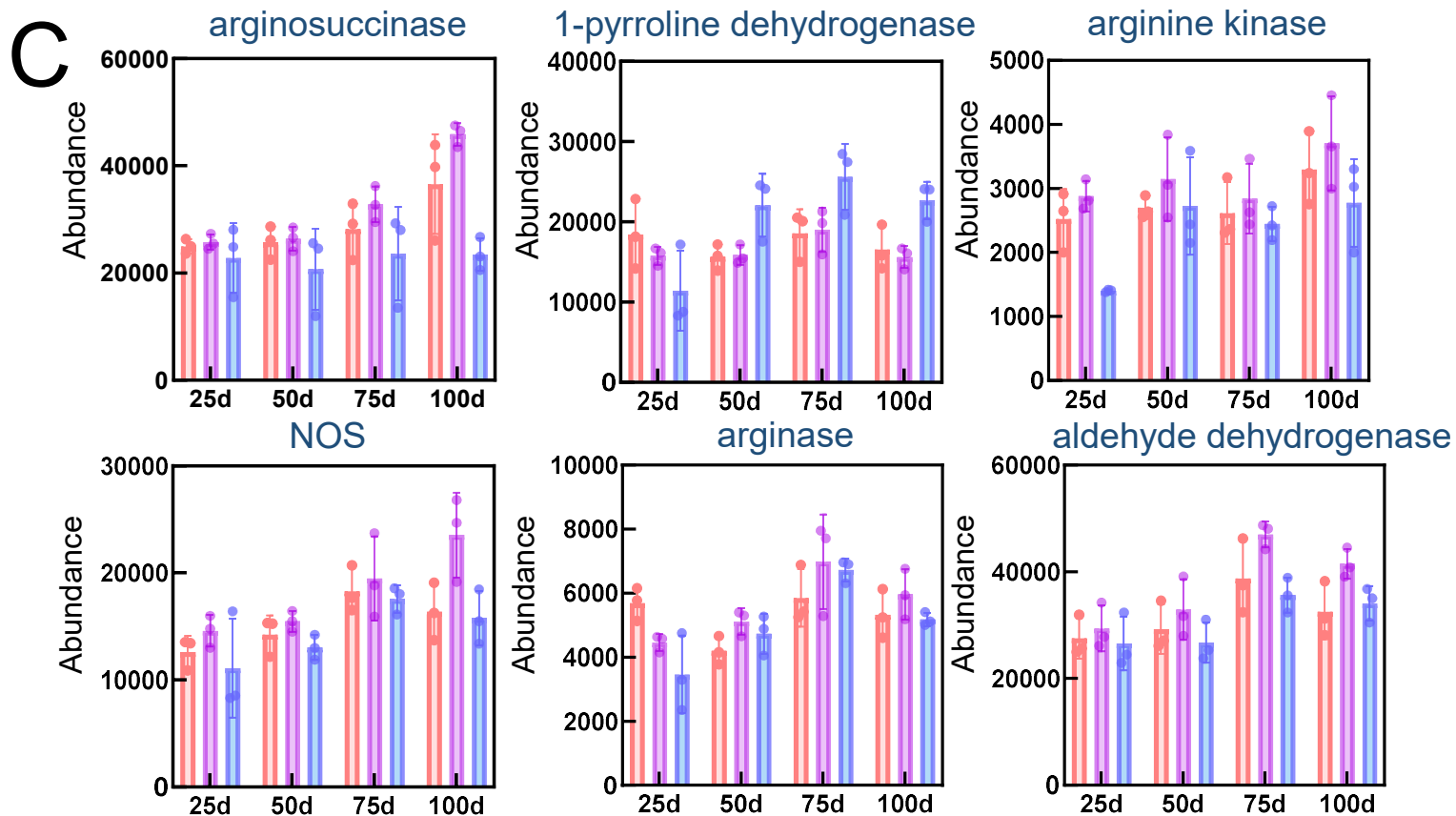

Supplement: Supplementary_wrad017 [file supplementary_wrad017.zip › Figure.S16.pdf]

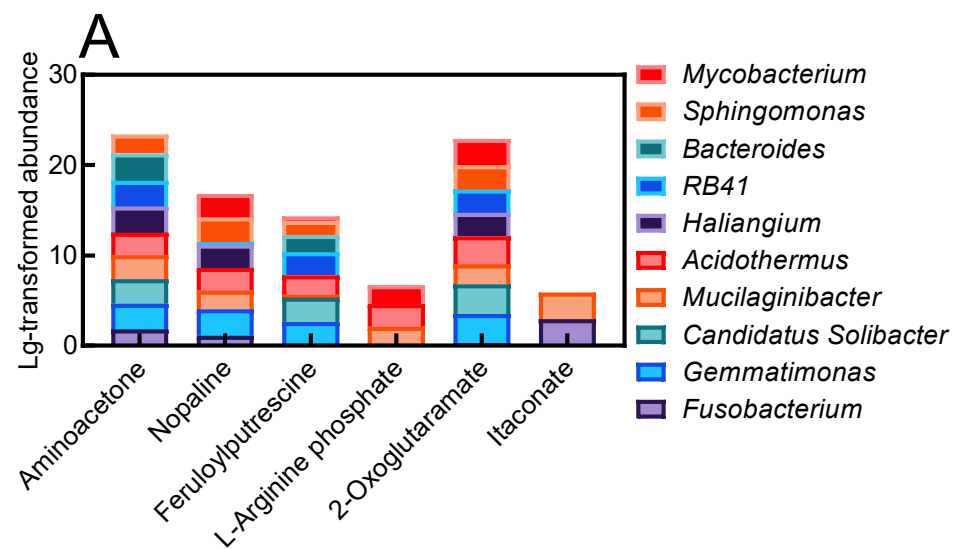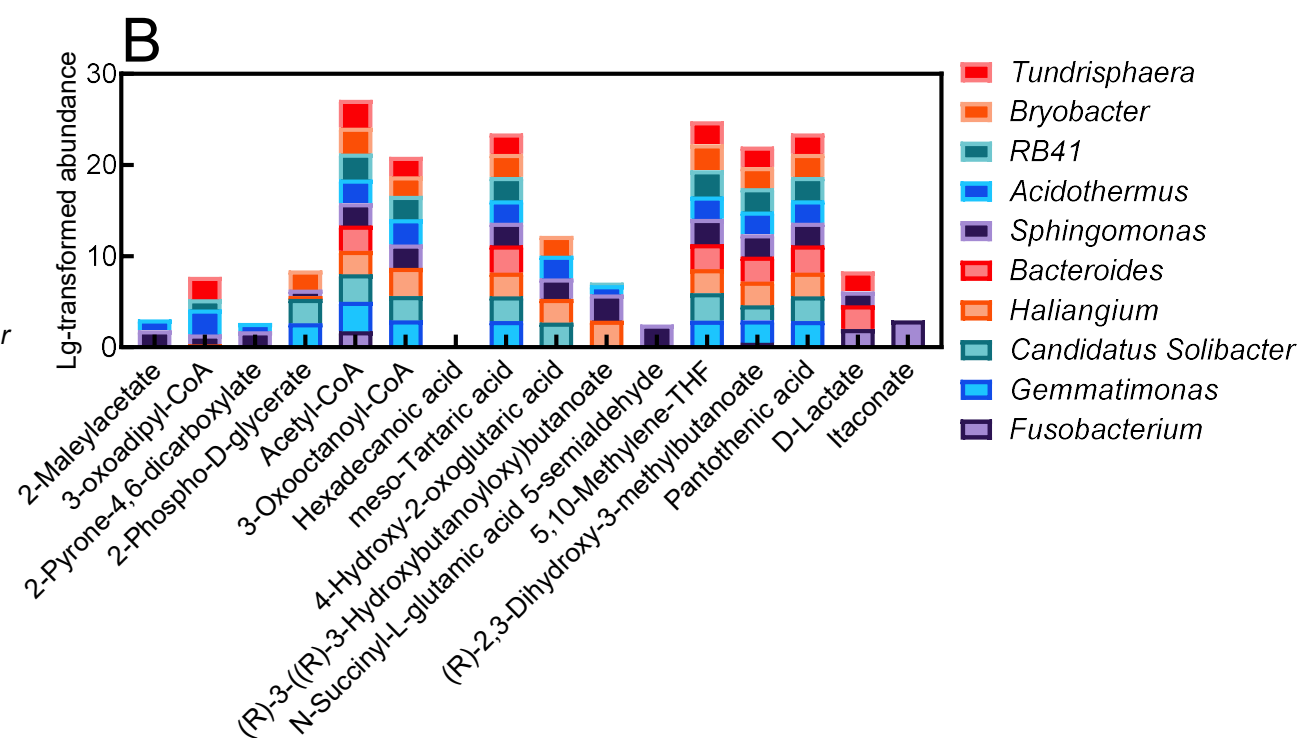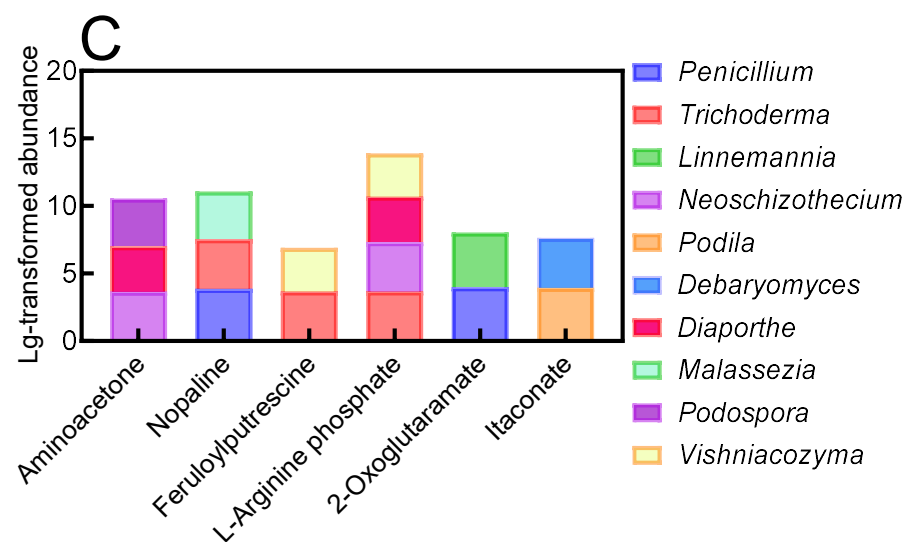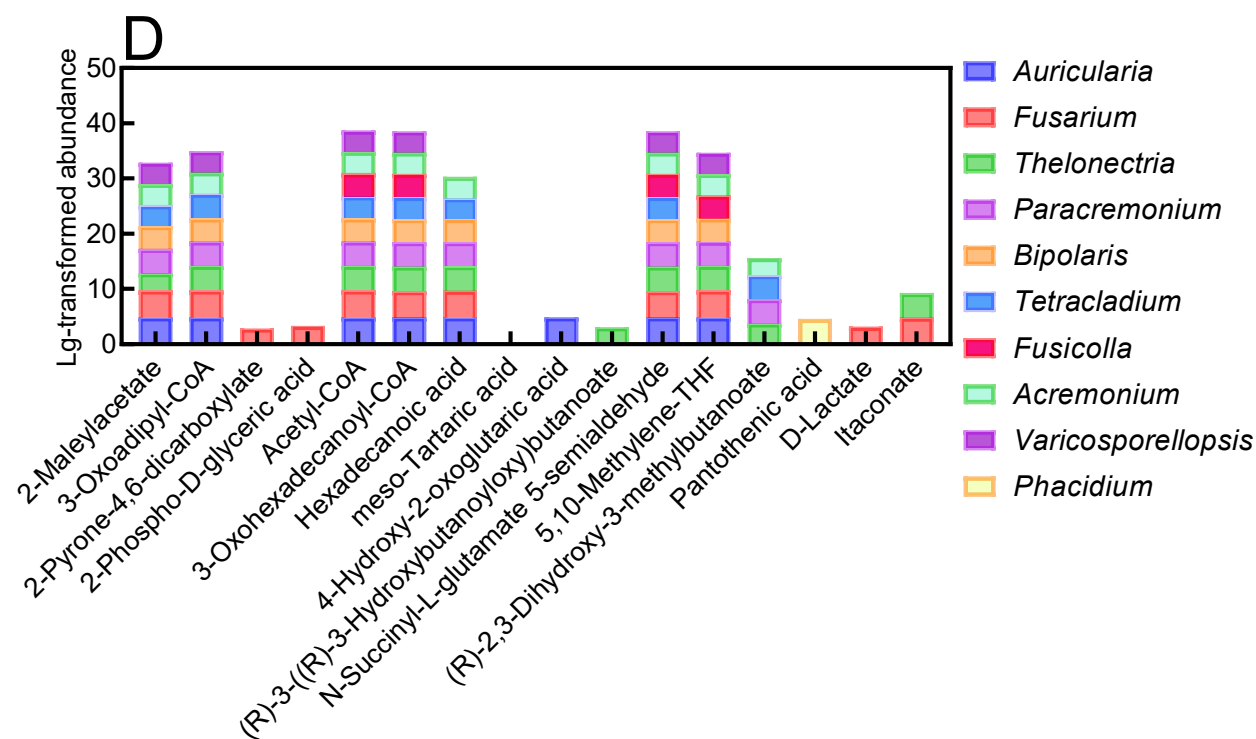

Supplement: Supplementary_wrad017 [file supplementary_wrad017.zip › Figure.S17.pdf]

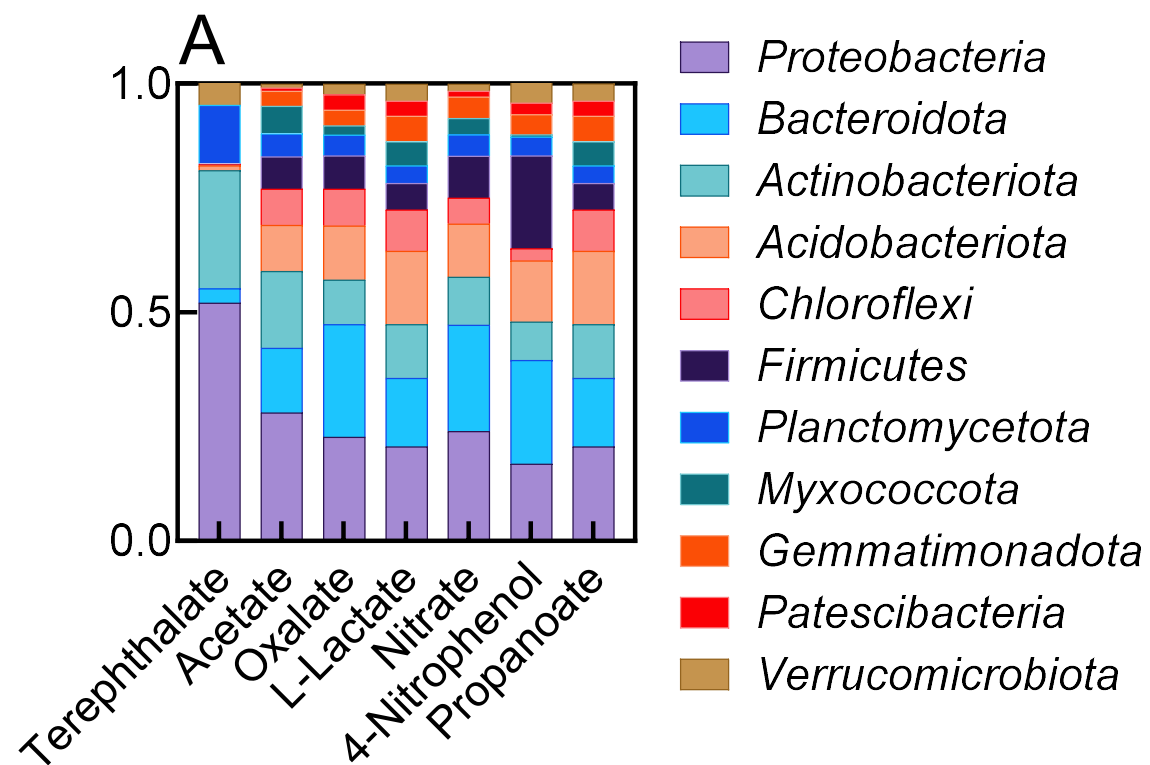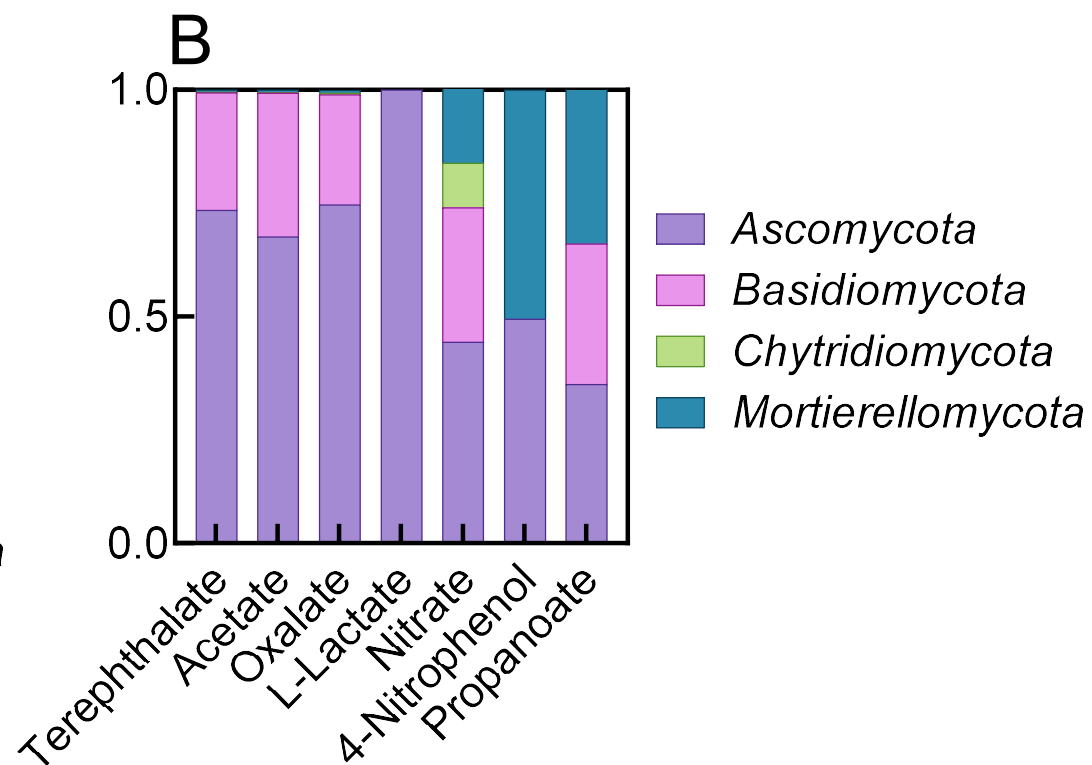

Supplement: Supplementary_wrad017 [file supplementary_wrad017.zip › Figure.S18.pdf]

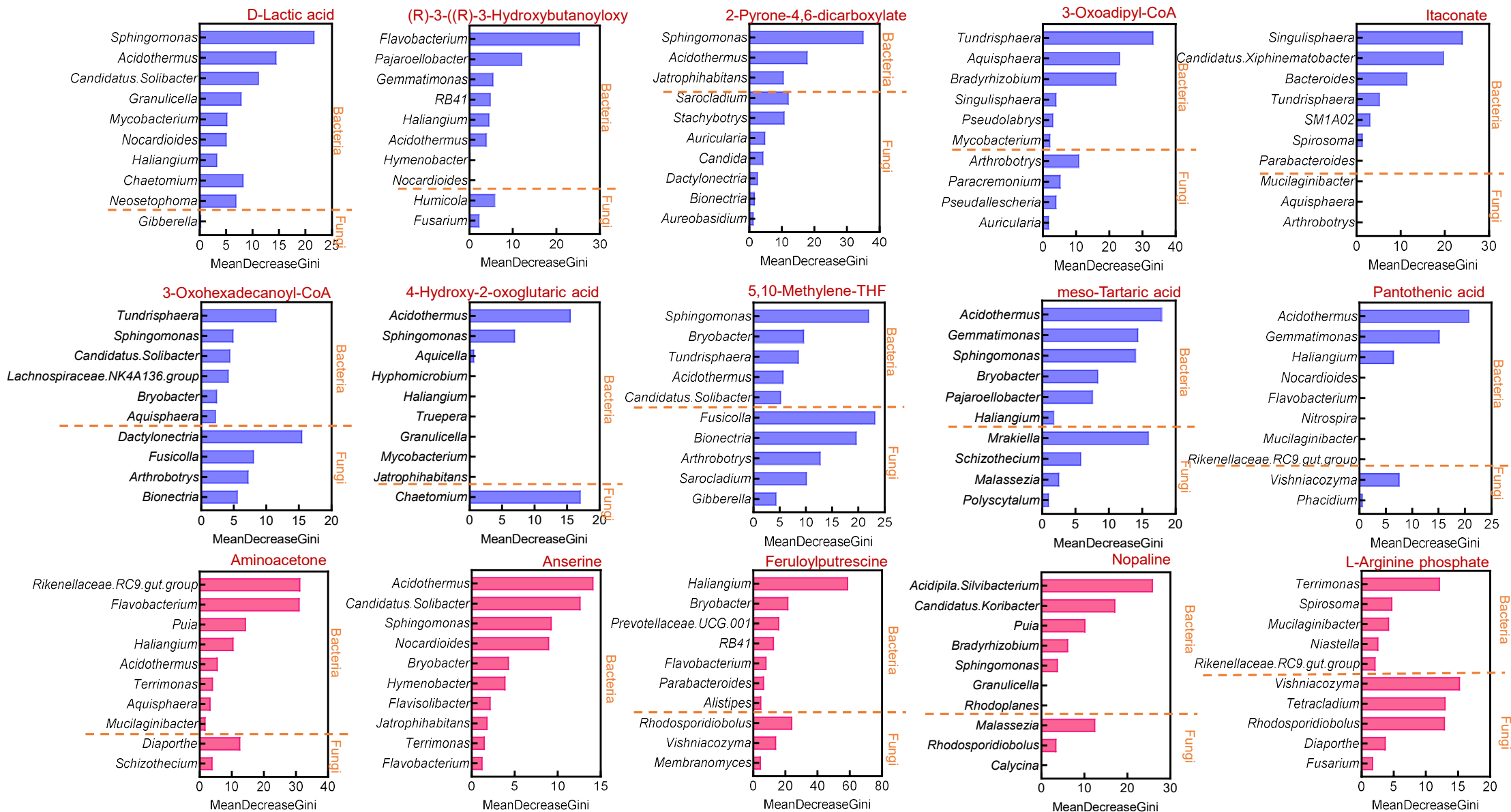

Supplement: Supplementary_wrad017 [file supplementary_wrad017.zip › Figure.S19.pdf]

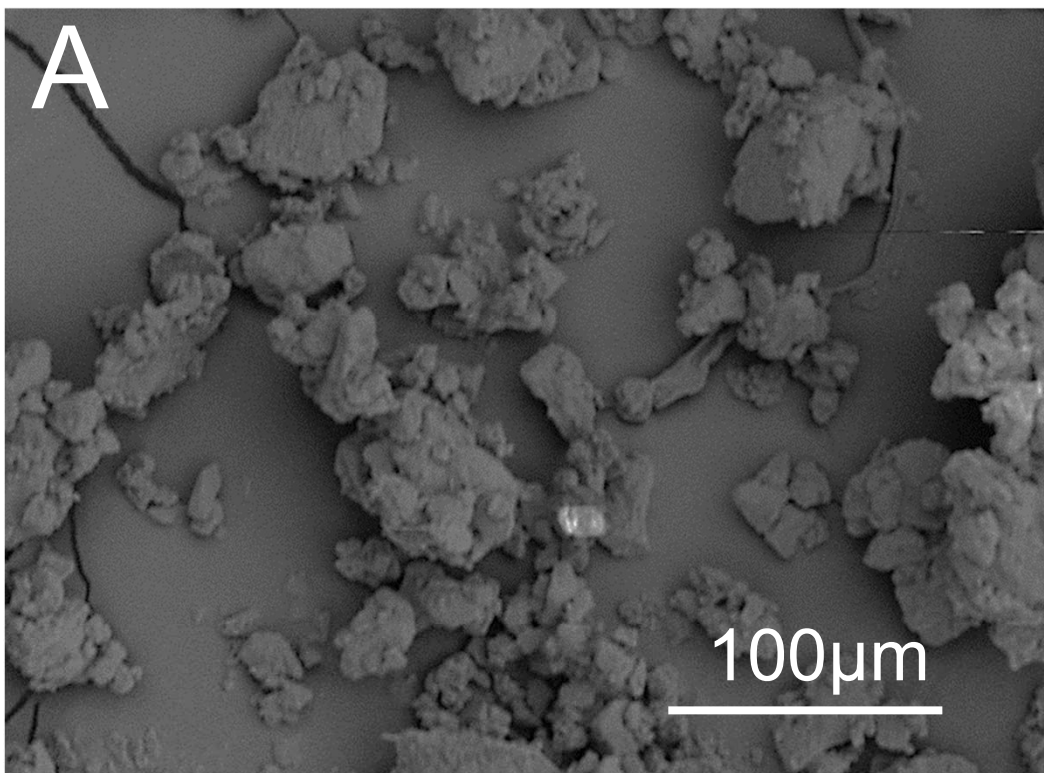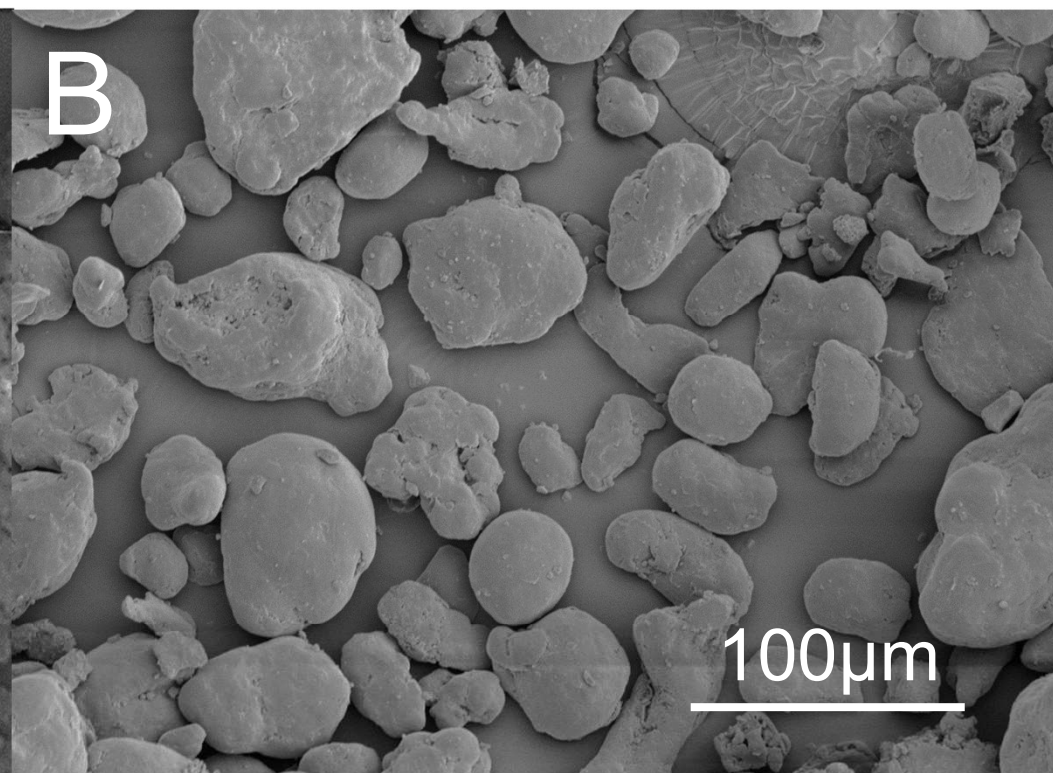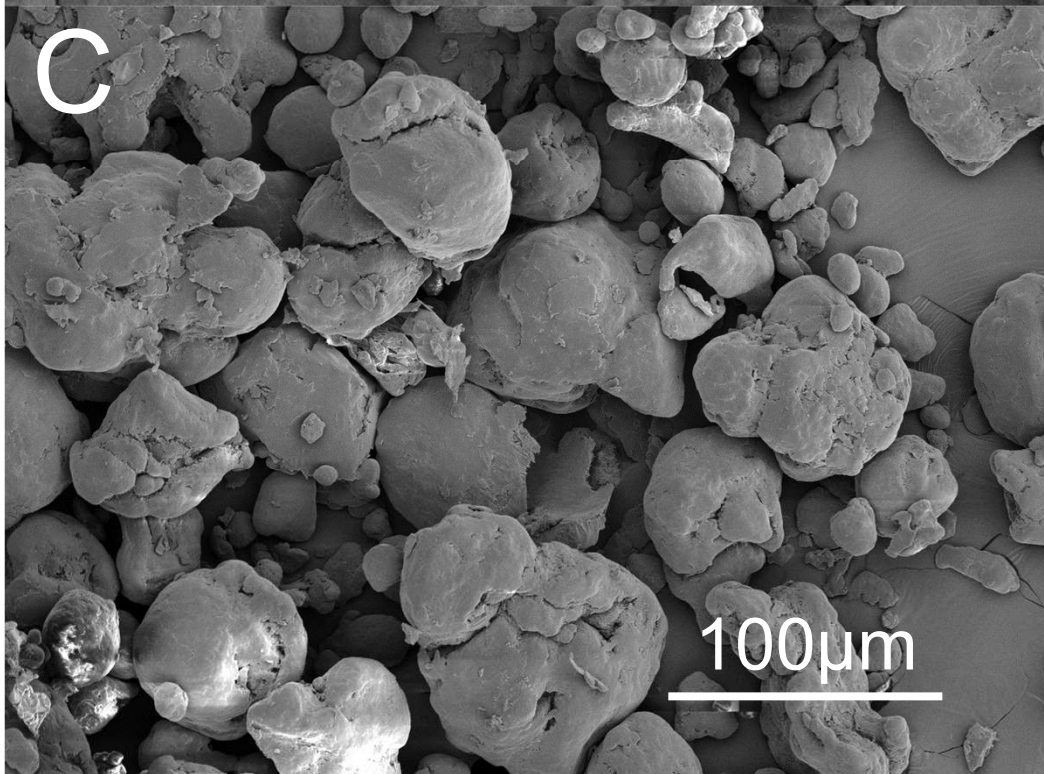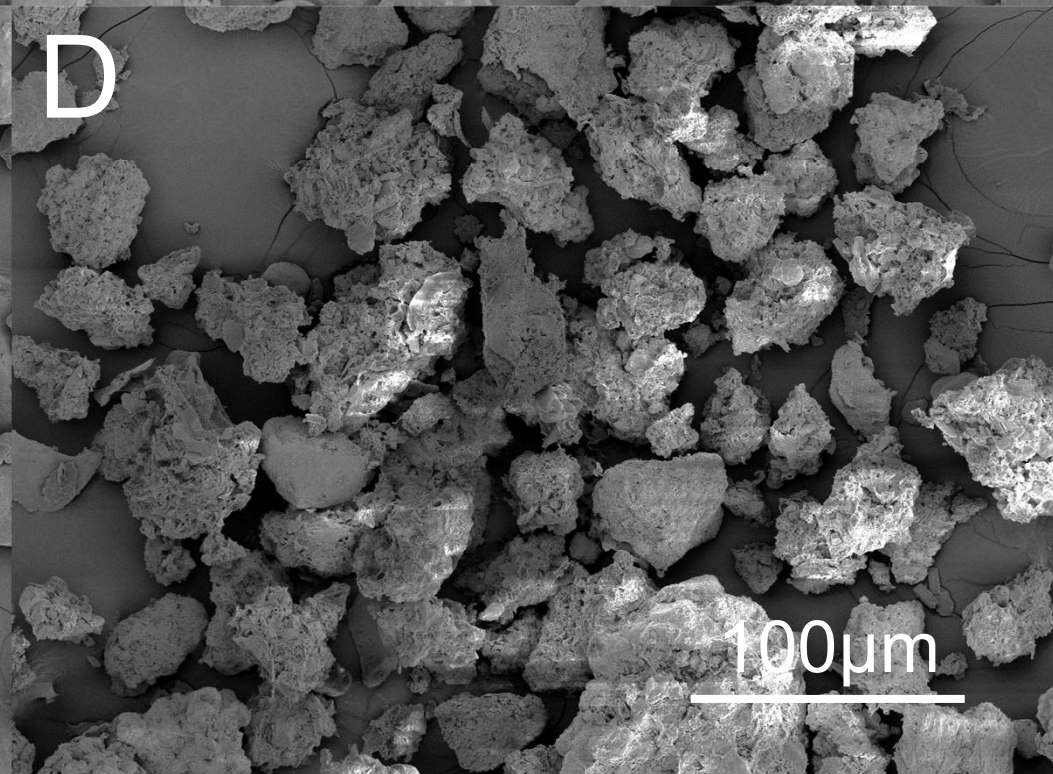

Supplement: Supplementary_wrad017 [file supplementary_wrad017.zip › Figure.S2.pdf]

## PLA

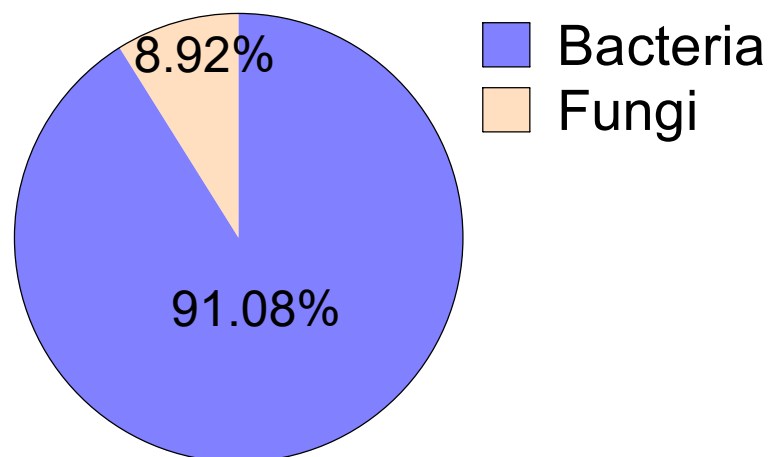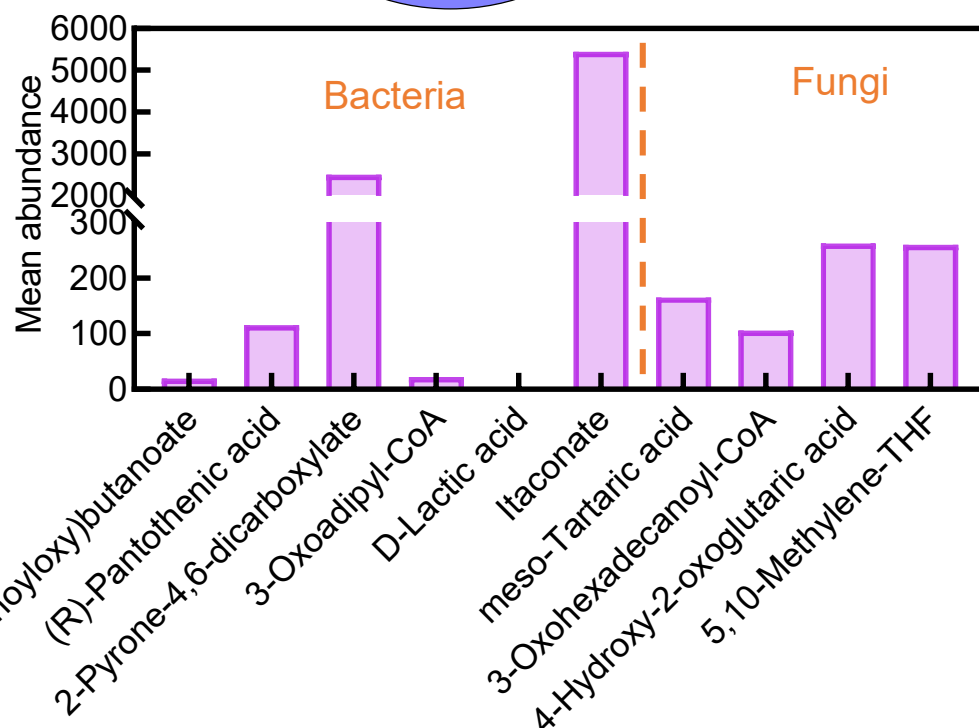

## PE

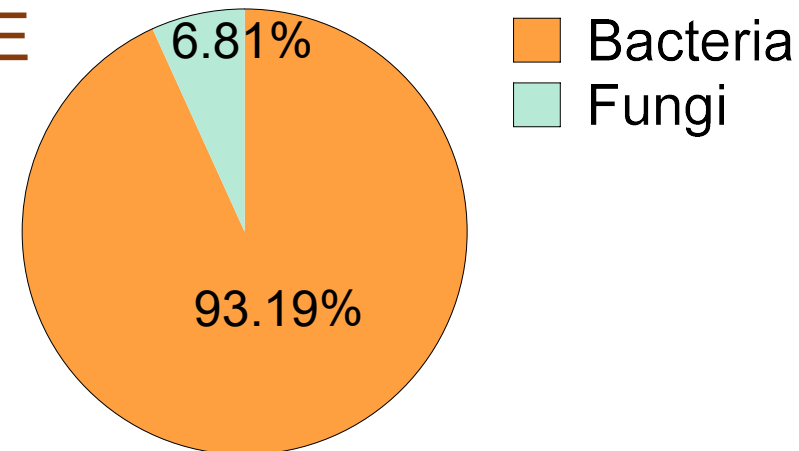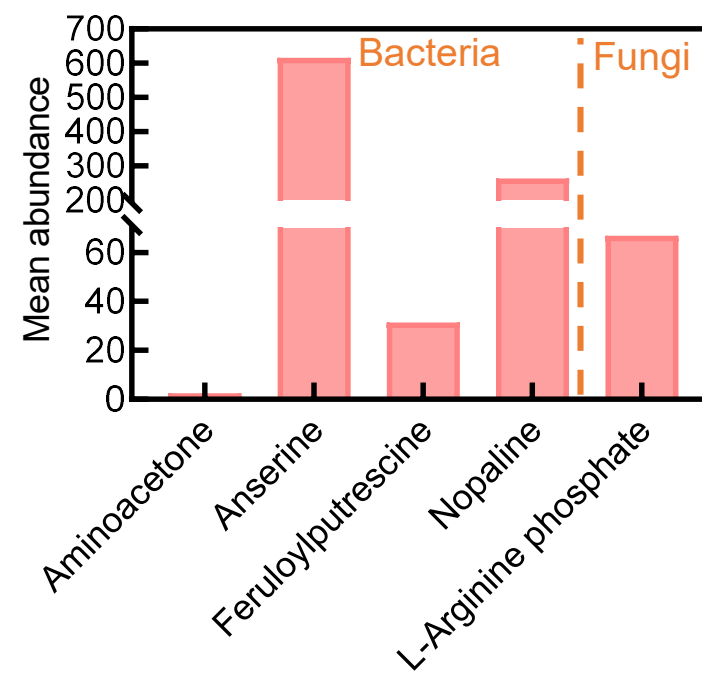

Supplement: Supplementary_wrad017 [file supplementary_wrad017.zip › Figure.S20.pdf]

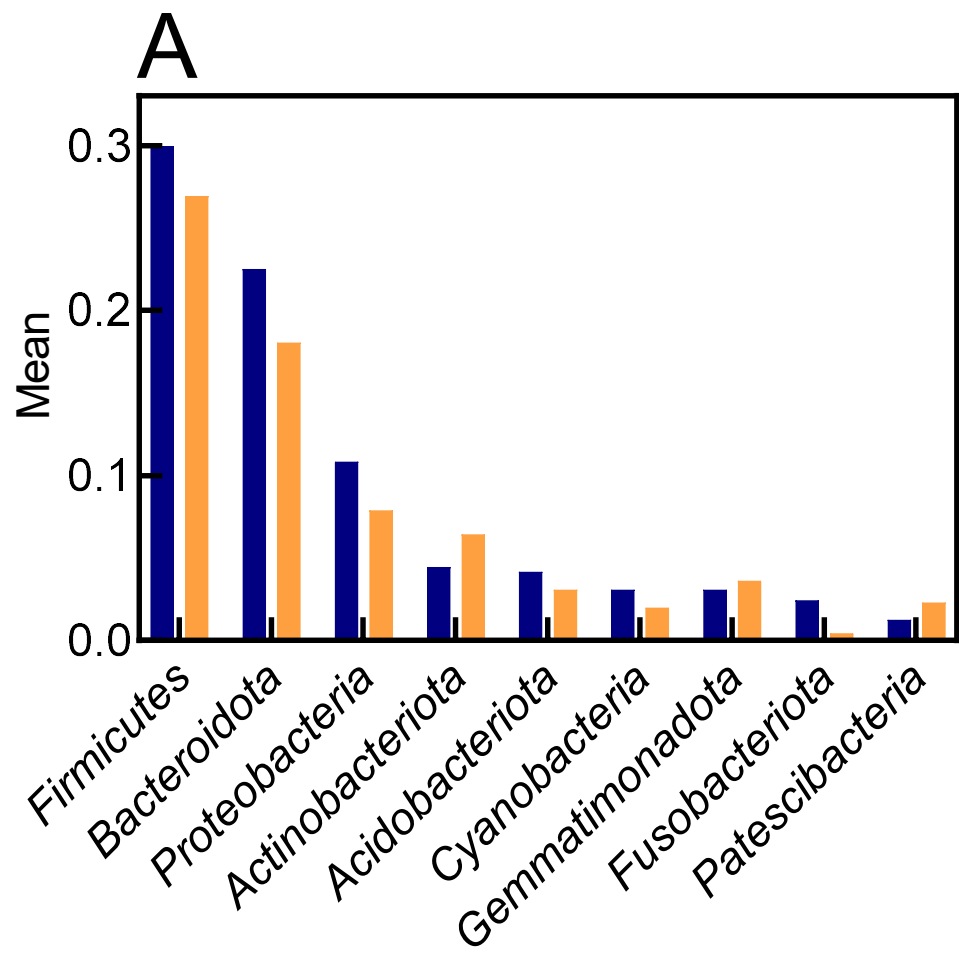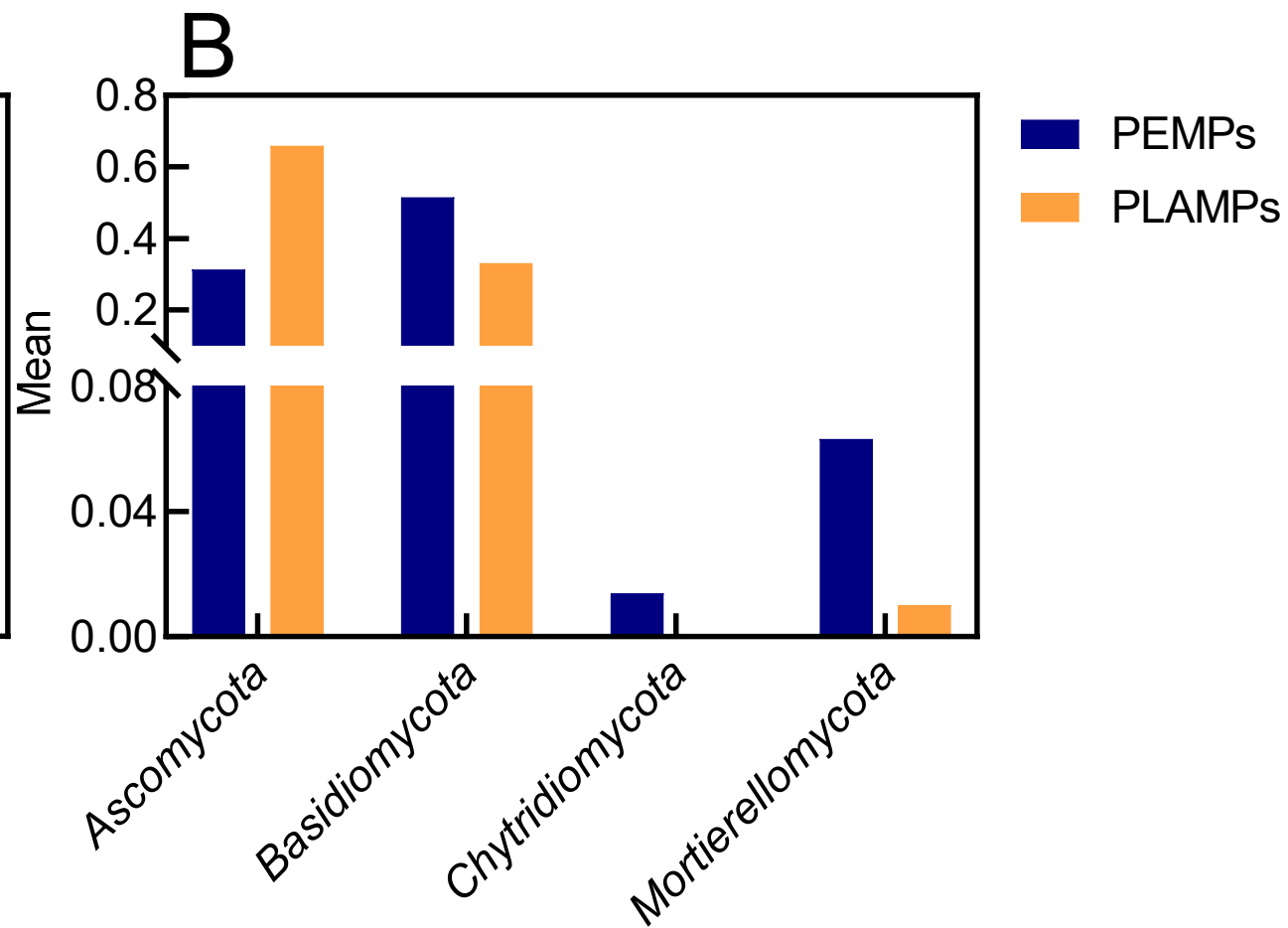

Supplement: Supplementary_wrad017 [file supplementary_wrad017.zip › Figure.S21.pdf]

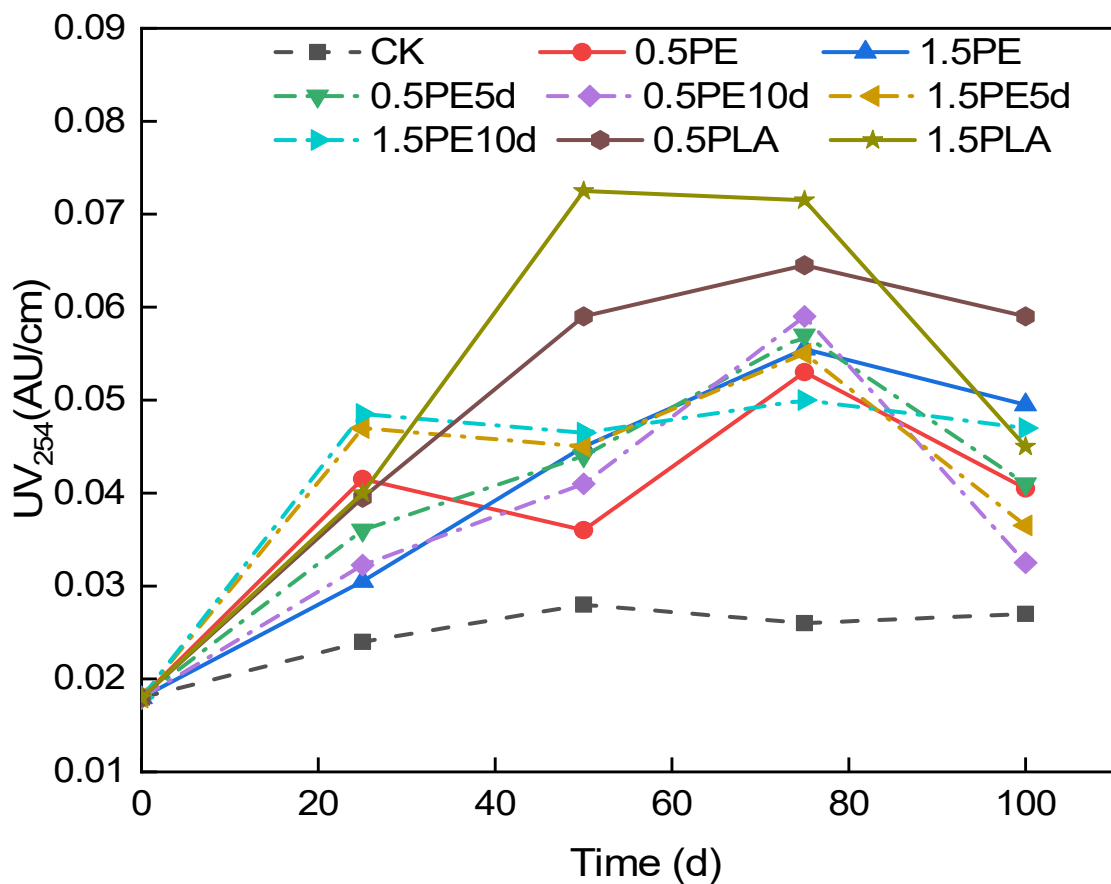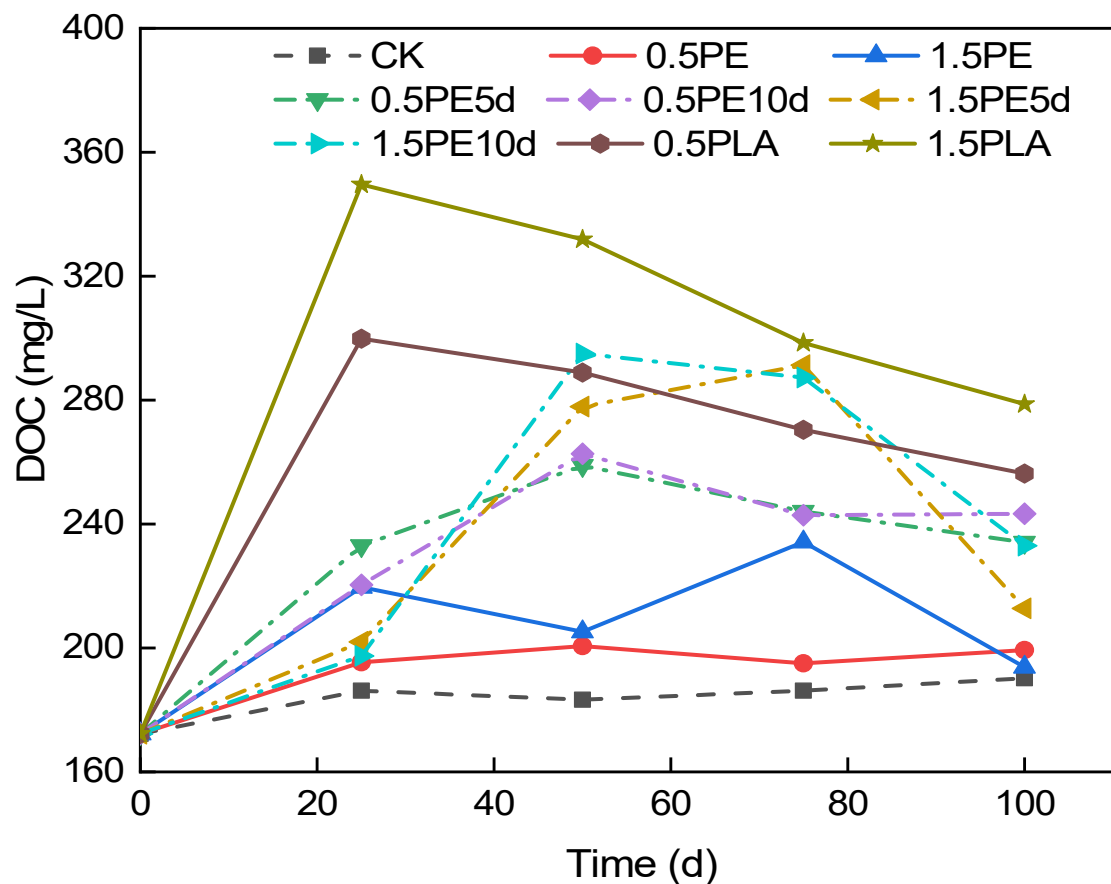

Supplement: Supplementary_wrad017 [file supplementary_wrad017.zip › Figure.S4.pdf]

25d

50d

75d

100d

0.5PE

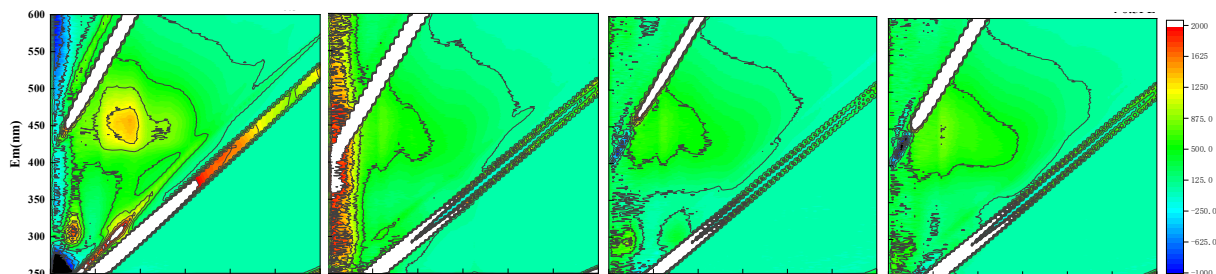

0.5PE5d

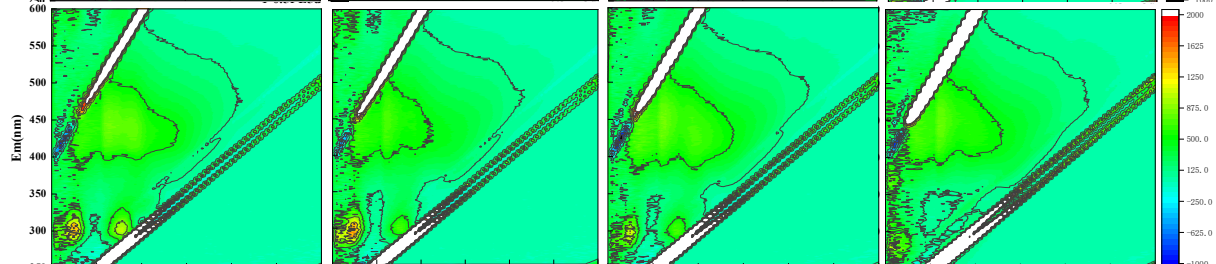

0.5PE10d

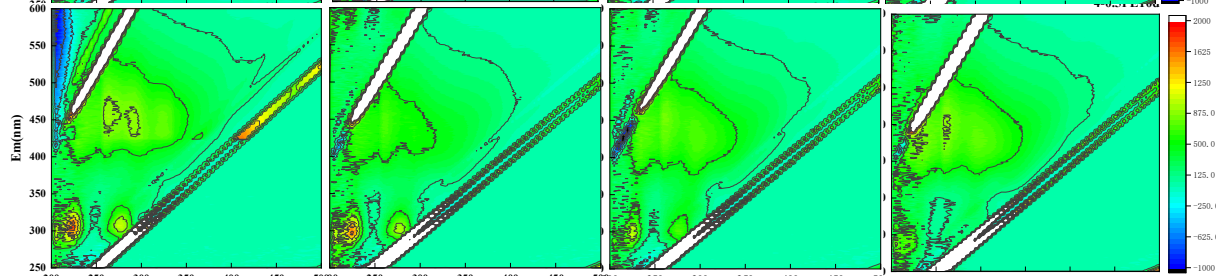

1.5PE

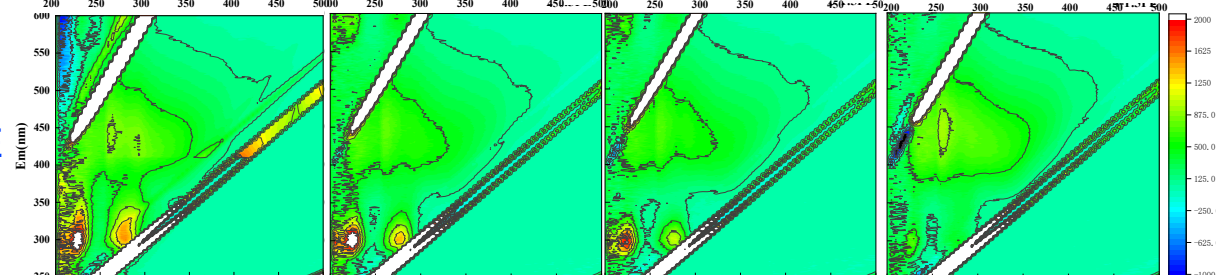

1.5PE5d

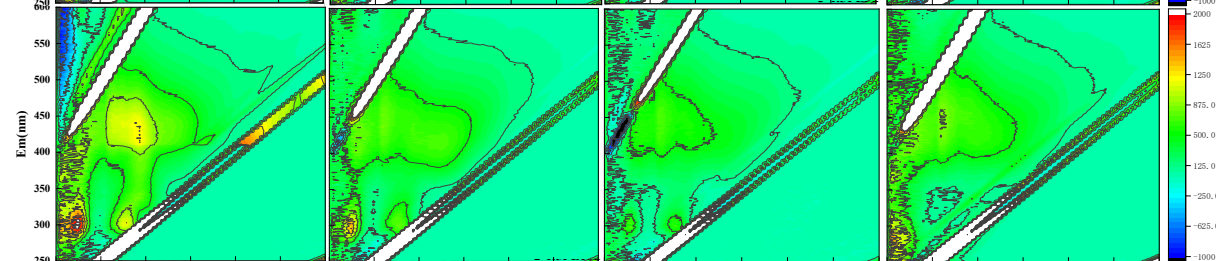

1.5PE10d

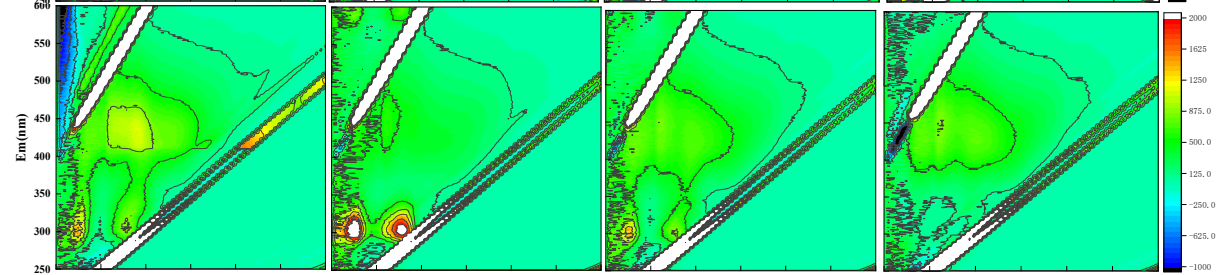

0.5PLA

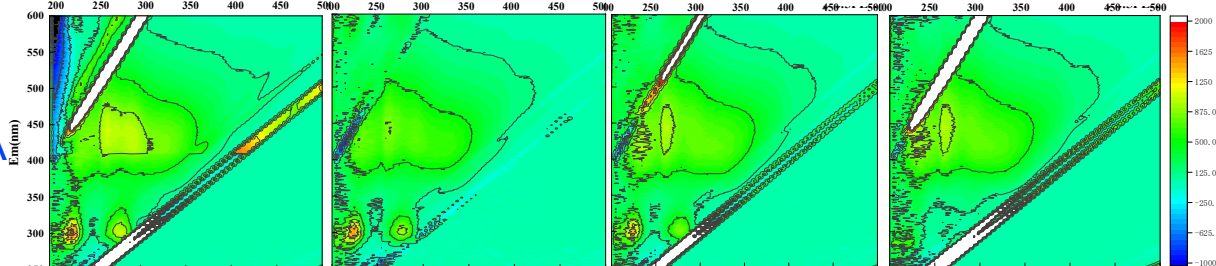

1.5PLA

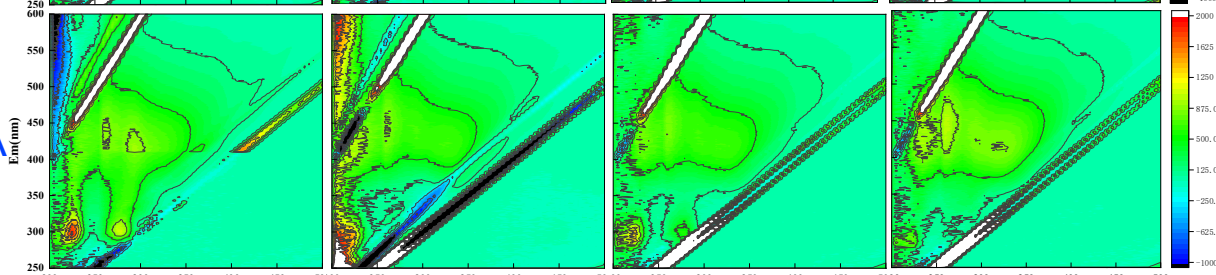

Supplement: Supplementary_wrad017 [file supplementary_wrad017.zip › Figure.S5.pdf]

PE  
(0, 0.5%, 1.5%)

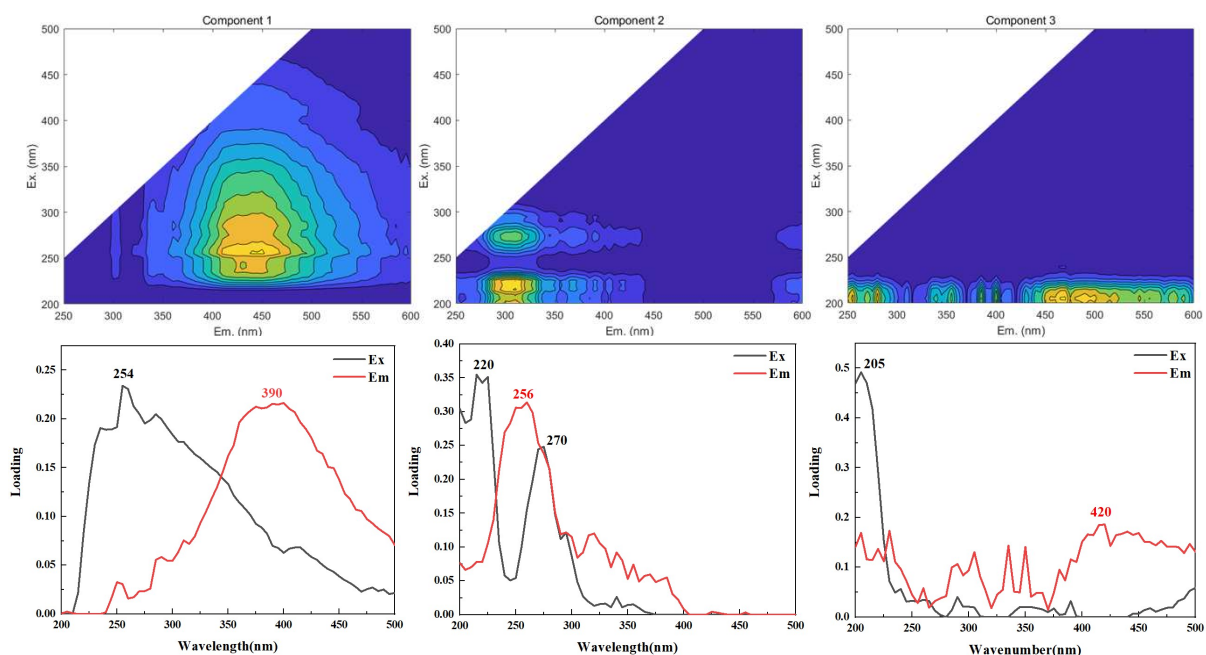

UV-aging PE

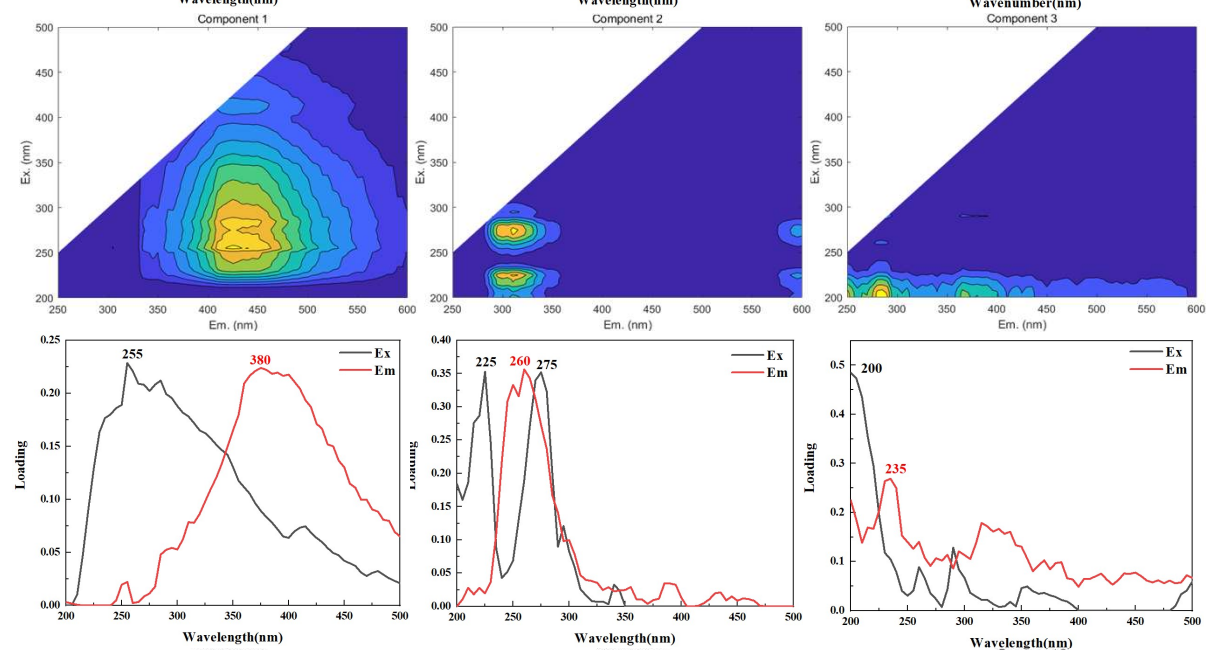

PLA

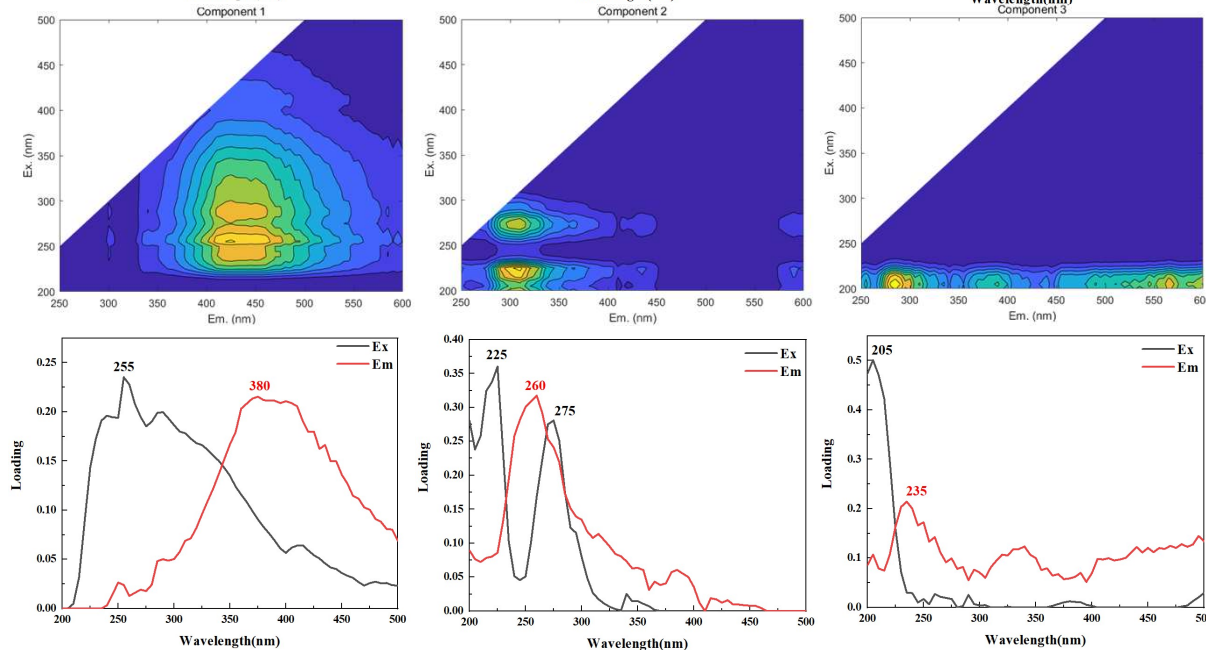

Supplement: Supplementary_wrad017 [file supplementary_wrad017.zip › Figure.S6.pdf]

**A**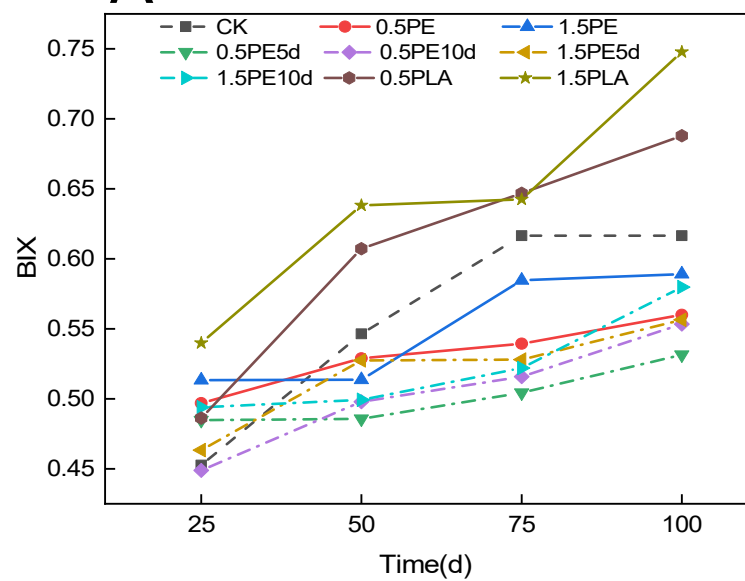**B**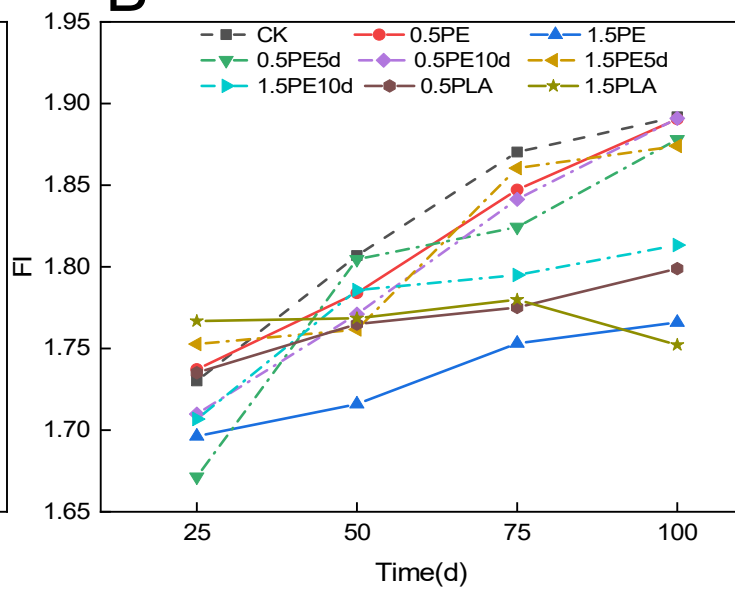**C**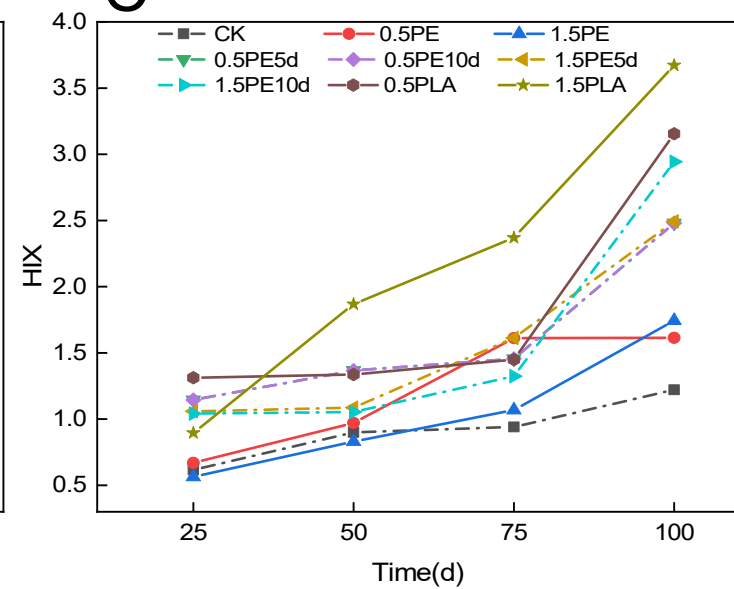

Supplement: Supplementary_wrad017 [file supplementary_wrad017.zip › Figure.S7.pdf]

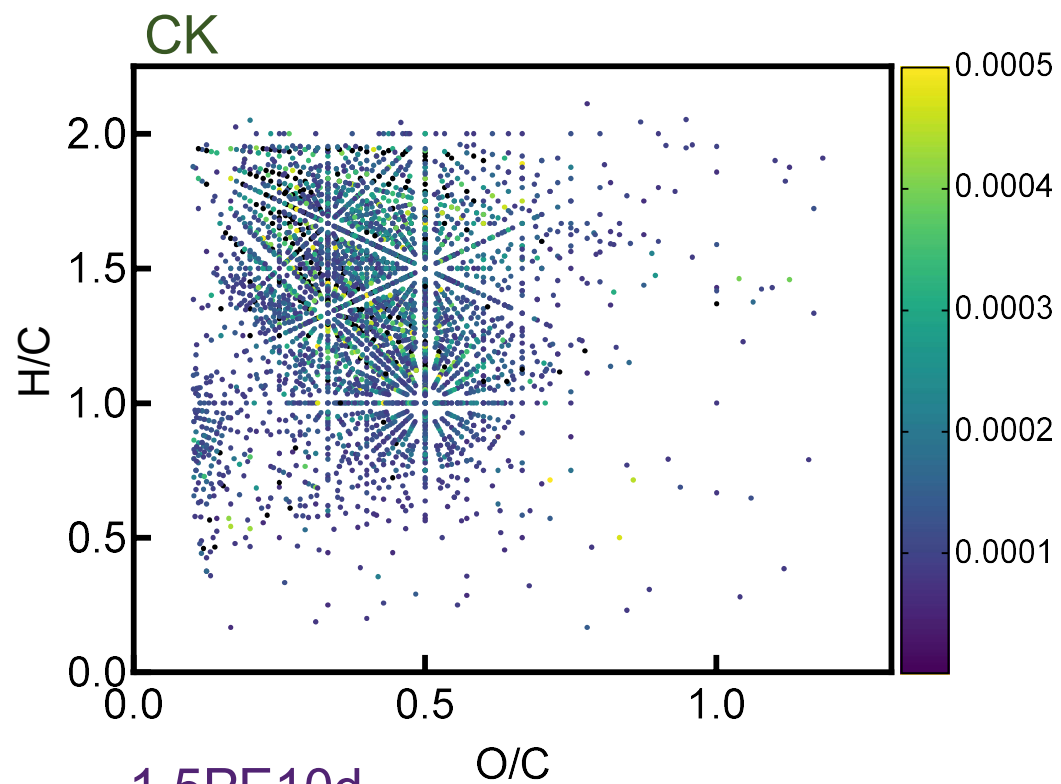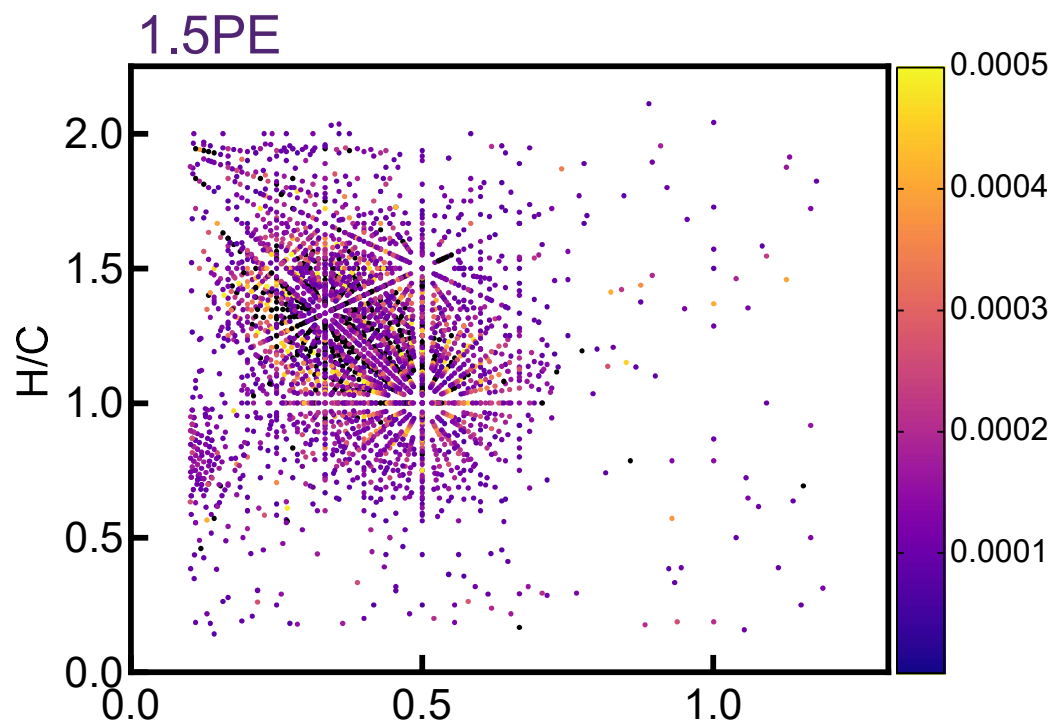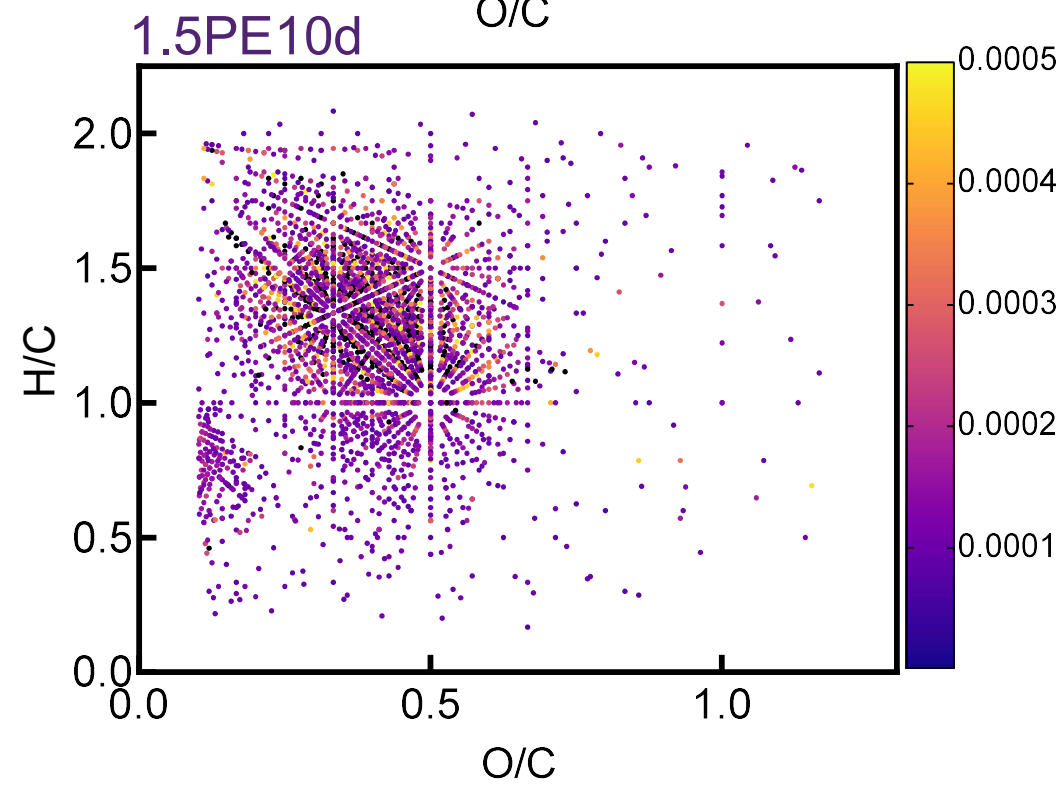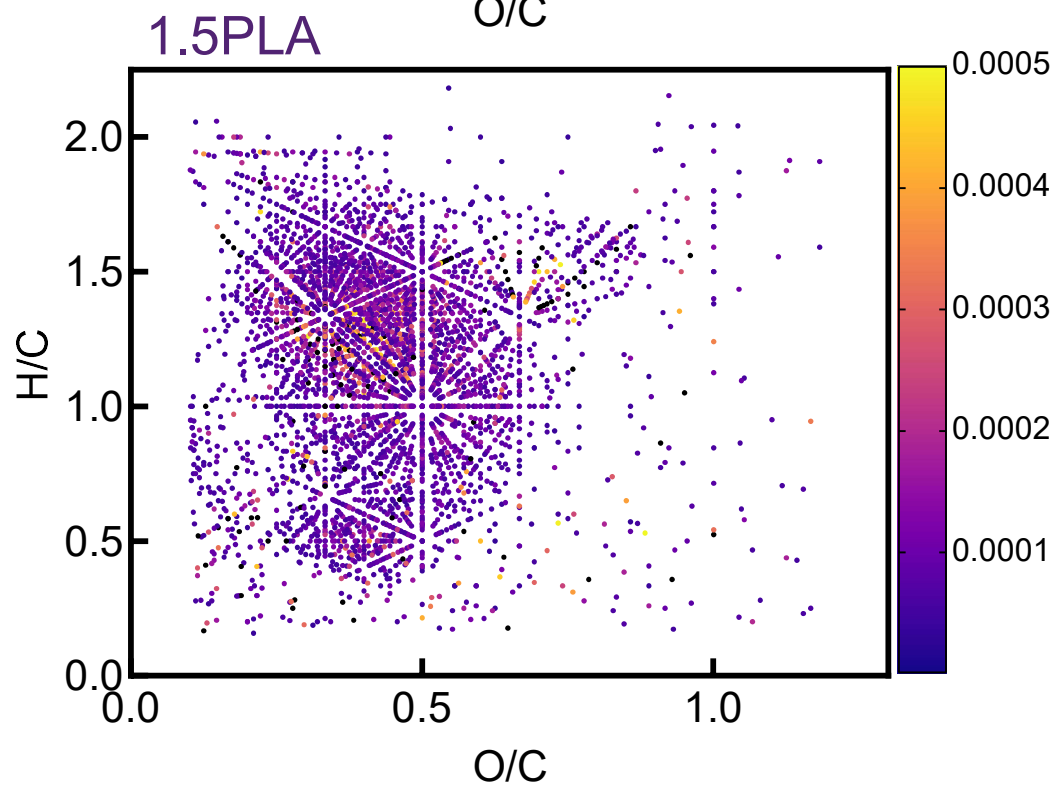

Supplement: Supplementary_wrad017 [file supplementary_wrad017.zip › Figure.S8.pdf]

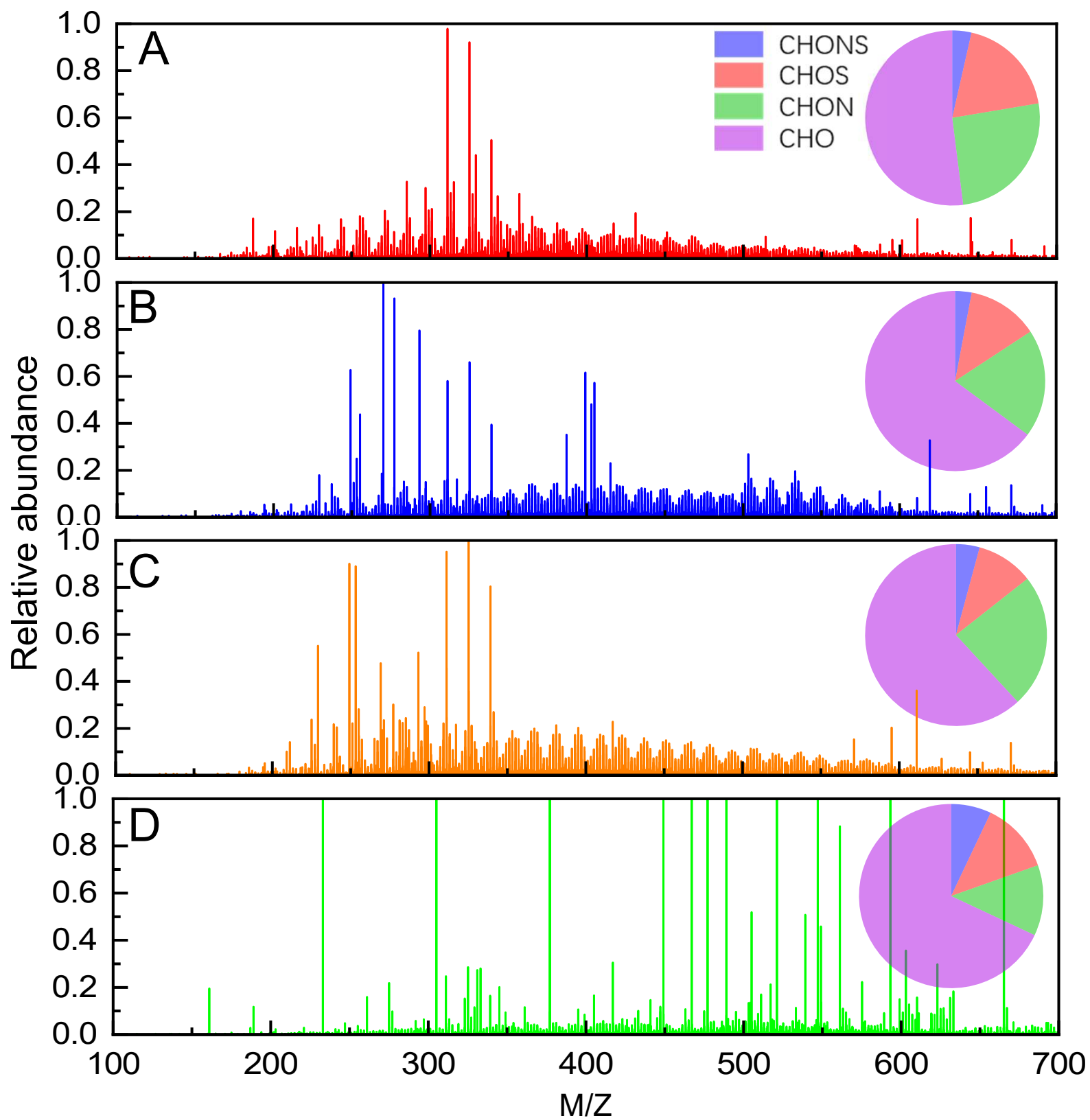

Supplement: Supplementary_wrad017 [file supplementary_wrad017.zip › Figure.S9.pdf]
